# The role of convergent ion channel pathways in microglial phenotypes: a systematic review of the implications for neurological and psychiatric disorders

**DOI:** 10.1038/s41398-018-0318-0

**Published:** 2018-11-29

**Authors:** Laiana A. Quagliato, Antonio E. Nardi

**Affiliations:** Laboratory of Panic and Respiration, Institute of Psychiatry, Federal University of Rio de Janeiro, Rua Ataulfo de Paiva 135s. 609, 22440-901 Rio de Janeiro, Brazil

## Abstract

Increases in the activated state of microglia, the main neuroimmune cells, are widely reported in the brains of patients with neurological and psychiatric disorders. Microglia transform from the resting to the activated state by sensing their environment, aided by a variety of ion channels. To examine the effect of ion channels on microglial phenotypes, we conducted a systematic review of immunohistochemical analyses of these neuroimmune cells in animal models following administration of ion channel antagonists, compared to control conditions. A systematic search of the PubMed and Web of Science electronic databases using the PRISMA and WHO methodologies for systematic reviews yielded 15 original peer-reviewed studies. The majority (13 out of 15) of these studies reported a decrease in microglial activated state after ion signaling pharmacological blockade. The studies provide evidence that acute administration of ion channel antagonists leads to a reduction in microglial activation in rodent brains in the models for epilepsy, Parkinson’s disease, inflammation, pain, ischemia, and brain and spinal cord injury. Future research should explore microglial-specific druggable targets for neurological and psychiatric disorders. The investigation of acute and chronic administration of ion channel antagonists in microglial phenotypes in primates and the development of microglia-like cells derived from human stem cells could be valuable sources in this direction.

## Introduction

Microglia, the resident immune cells of the central nervous system (CNS), can differentiate into distinct phenotypes, including resting and activated cells^1^. In vivo resting microglial cells exhibit a ramified morphology, characterized by several branched processes arising from an elongated and flattened cell body. Microglial cells undergo shape changes following activation, i.e., cells transform from ramified to ameboid morphology in response to neuronal injury and during inflammation or infection^1^. Activated microglia are capable of proliferation, migration, and antigen presentation and release a variety of substances that can be either neuroprotective or neurotoxic^1^.

Evidence indicates that changes in microglial morphology depend on the cells sensing the environment by repeatedly extending and retracting their processes, but the factors regulating microglial surveillance are unknown^2^. Microglia interact with their environment with the aid of a complicated ensemble of transporters and ion channels^3^. The latter include purinergic metabotropic P2Y receptors and ionotropic P2X receptors, the transient receptor potential (TRP) channels such as TRPC6, and the K^+^ channels kir 2.1, KV 1.3 and KCa 3.1^1,4^. The variety of channels expressed by microglia shows complex spatiotemporal patterns according to changes in the immune cells’ microenvironment, which may contribute to the different phenotypes expressed by microglia^5^. Furthermore, these various molecules play different roles in microglial function. For instance, K^+^ channels such as kir, KV, and KCa act by regulating microglial membrane potential^6^. TRP channels are associated with microglial activation^1,7^, and the purinergic receptors P2X and P2Y are related to microglial surveillance and phagocytic activity^8^. Additionally, all the channels may maintain the resting potential of microglia and thus contribute to microglial ramification and continuous surveillance of the brain via process motility^1^.

In recent decades, an increasingly compelling body of evidence has emerged linking microglial activation to neurological and psychiatric disorders. Broadly, this evidence stems from the observations that microglial surveillance plays an important role in monitoring synaptic function and determining brain connectivity^9,10^. During postnatal development, synapses that are to be pruned become tagged with complement molecules and are thus removed by microglia^11^. Disruption of this system may lead to altered CNS connectivity, generating excess excitatory synapses that may be involved in the pathogenesis of various disorders, such as epilepsy^12^ and autism^13^. Furthermore, microglial cells release different molecules that are potentially implicated in an excitatory-inhibitory imbalance, which may also contribute to the pathogenesis of psychiatric and neurological disorders^14^. Based on the above, we review the evidence of current preclinical literature on ion signaling in microglial phenotypes, providing evidence for the role of ion channels in microglial state and identifying gaps in the literature to inform future research.

### Our primary outcome

- Does ion channel pharmacological blockade modify microglial phenotype?

### Our secondary outcomes

- How does interaction between microglial ion channels occur?
- Can ion signaling contribute to the development of neurological and psychiatric disorders?

## Materials and methods

The systematic search was conducted in PubMed and Web of Science, covering articles published up to 31 December 2017. The search protocol was developed based on Preferred Reporting Items for Systematic Reviews and Meta-Analyses (PRISMA) and World Health Organization (WHO) Review Protocol Template Guidelines where applicable for this systematic review, as provided in Supplementary Materials Section 1. We also manually checked references cited in the systematically searched articles. To avoid publication bias, non-english language studies and gray literature (for example, conference abstracts) were included. A broad but highly structured search strategy was used, based on the PICOS framework. The study population was microglia, the intervention/exposure was ionic channel antagonism, comparison was with absence of ion channel blockade, outcome was resting versus activated microglia, and study design included any type of design. Keywords for the search included various combinations of terms for microglia and the nervous system, including both historical and contemporary ion channel names. A full list of terms used for the search strategy can be found in Supplementary Materials Section 2.

### Study selection

Studies were selected for data extraction and analysis based on the following inclusion criteria: (1) ion channel antagonists administered in vivo and (2) studies evaluating microglial activated or resting state. Exclusion criteria were (1) studies that lacked a baseline condition or control group, (2) studies that did not report original data, (3) studies without immunohistochemical analysis of microglia, (4) studies evaluating genetic ionic channel deficiencies, (5) studies that investigated only in vitro ion channel blockade. Due to the highly reactive nature of microglia, which substantially alters their morphology and functional properties when exposed to culture conditions^15^, in vitro studies should be interpreted with caution.

### Data extraction

A standard data extraction template adapted from the Cochrane checklist of items (Supplementary Materials Section 3) was used. As the type of outcome reporting was extremely heterogeneous, results were reported as higher, lower, or unchanged for ion channel antagonism relative to control conditions as identified. Meta-analyses and other summary statistics were not used because of the wide variation between studies in assessment techniques and brain regions examined.

### Quality assessment

This systematic review was performed according to preferred reporting items for systematic reviews and meta-analyses (PRISMA) guidelines^16^ (Supplementary Materials Section 4). Quality assessment used SYRCLES Bias Tool^17^ (Supplementary Materials Section 5).

## Results

**Fig. 1:**
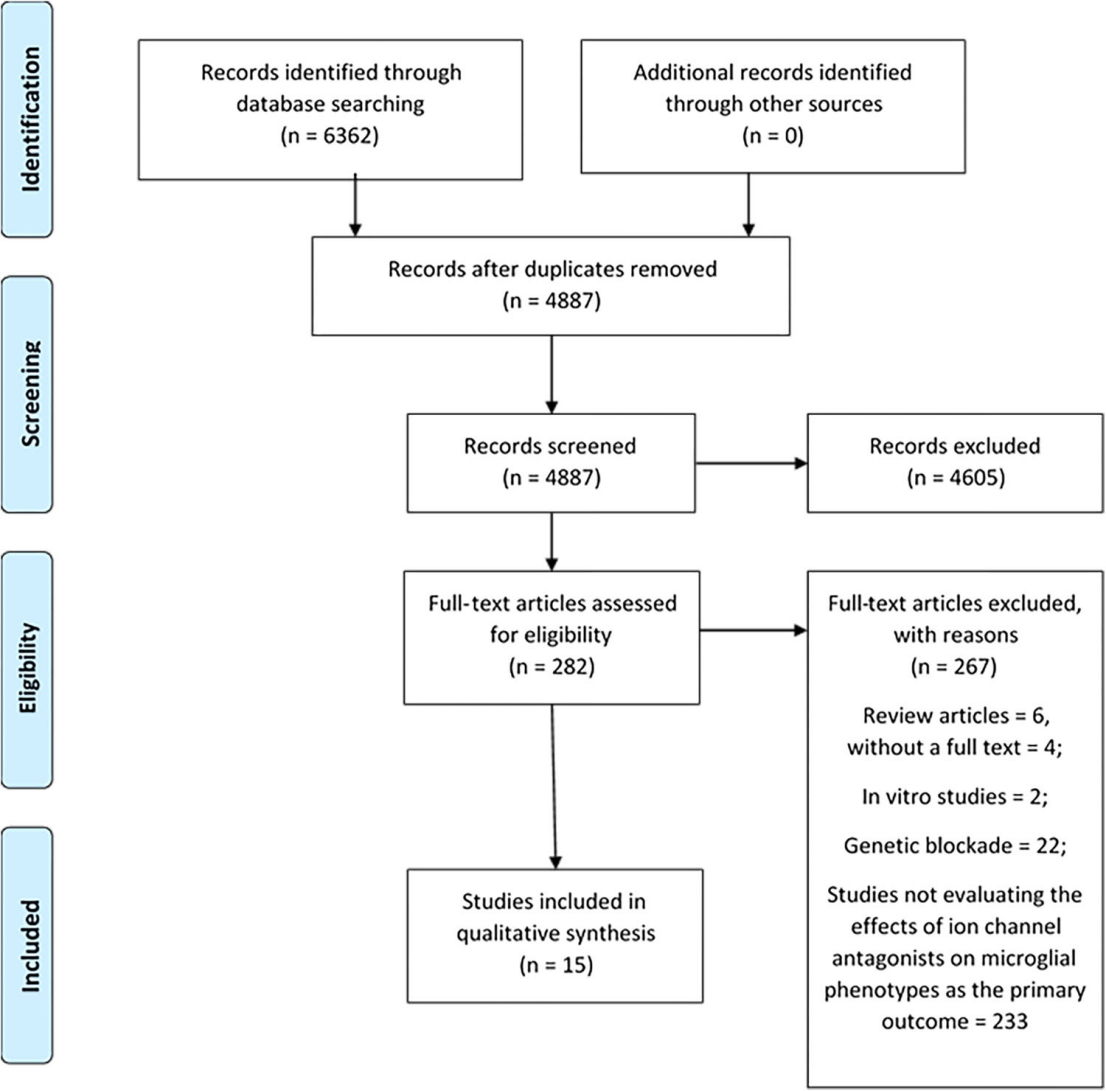
**PRISMA Diagram**

The literature search identified 6362 potentially relevant articles for initial screening. Duplicates (*N* = 1475) were identified using a function in Endnote and confirmed by manual screening of the titles. A total of 4605 studies were excluded from initial assessment of titles and abstracts. In all, 282 abstracts were classified for possible inclusion, and their full texts were obtained. In all, 267 papers were excluded from further analysis. In all, 282 full texts were reviewed, of which 15 met the inclusion criteria for our systematic review (see Fig. 1 for a PRISMA diagram of the literature search). Table 1 summarizes the included studies. Additional data regarding the included studies can be found in Supplementary Materials Section 6. Reasons for the exclusion of studies can be found in Supplementary Material Section 7.

**Table 1:** Studies of microglial phenotypes following administration of ion channels antagonists

Condition studied	Receptor	Study	Animal model	*n*	Antagonist/deficiency	Duration of intervention	Intervention dose	Timepoint for outcome measurement	Immunoreactivity	Region of interest	Change in ion/neurotransmitter channel measure after injury/lesion	Change in microglial activation after antagonist infusion compared to control condition
Epilepsy	P2X7	Choi et al. 2012^18^	Pilocarpine	30 × 37	OxATP	1 week	0,5 mmL/h	1, 4, and 8 weeks after SE	Iba-1	Hippocampus	na	↓
		Huang et al. 2017^19^	Corialactone	30/group	BBG; A-438079 and A-740003	30 min prior to and 60 min post injection	BBG: 1, 5, 10 µg; A-438079 and A-740003: 10 µG	0, 8, 24, 2 days, 4 days, 1 week, 2 weeks after SE	Iba-1	Hippocampus	↑	↓
	TRPC6	Lee et al. 2014^20^	Pilocarpine	12/group	Hyperforin	1 week	6 µM	3 days	Iba-1	Piriform cortex	↑	↓
Ischemia	P2X7	Yu et al. 2013^21^	Four-vessel occlusion method	5/group	BBG and A-740003	3 days	BBG: 50 mg/Kg and A-740003: 100 mml/Kg	After 12 h, 24 h, 48 h, 4 days, 7 days of I/R injury	IB4	Hippocampus	↑	↓
		Chu et al. 2012^22^	Four-vessel occlusion method	4/group	BBG and OxATP and A-438079	Right before cerebral injury	BBG 10 µg, OxATP 1 µg, A-438079 3 µg	After 3 days of reperfusion	Iba-1	Hippocampus	na	↓
		Melani et al. 2006^23^	MCAo	14/group	RB2	5 min after sham operation or MCAo	100 mg/Kg	24 h after surgical procedures	OX-42	Striatum, hippocampus	↑	↑
	P2X4	Wixey et al. 2009^24^	Hypoxia–ischemia model	8 × 12	Minocycline	9 days	45 mg/kg i.p. 2 h post insult then 22.5 mg/kg i.p. every 24 h until day 9	10 days after the insult	Iba-1	Corpus callosum, cingulum	↑	↓
	k-atp channel	Ortega et al. 2012^26^	MCAo	15/group	Glibenclamide	6, 12, and 24 h after reperfusion	0.06 or 0.6 or 6 mcg	72 h after MCAo	IB4	Subcortical and cortical region	↑	-----
	P2Y12	Gelosa et al. 2014^26^	MCAo	6/group	Ticagrelor	10 min, and then 22 and 36 h after MCAo	3 mg/Kg or 30 mg/Kg	2 h after MCAo	Iba-1 and ED-1	Dorsal and ventral areas	↑	↓
TBI	P2X7	Liu et al. 2017^28^	Modified weight drop technique	6/group	A804598	5 days	A804598: 10 mg/Kg	6,12, 24 h	Iba-1	Cortex	↑	↓
Spinal cord injury	P2X4	Zhou et al. 2014^29^	Method of Decosterd and Woolf	4/group	Dexmedetomidine	14 days	40 mcg/kg	1, 3, 5, 7, 14 h after surgery	Iba-1	Spinal cord	↑	↓
Pain	P2X7	He et al. 2012^30^	Chronic constriction injury model	7/group	BBG	14 days	10 mcl	14 days after nerve injury	OX-42	Spinal cord	↑	↓
Parkinson	P2X7	Wang et al. 2017^31^	LPS injected to the right substantia nigra	12 × 6	BBG	15 days	50 mg/ Kg	15 days	Iba-1	Substantia nigra	↑	↓
	kir2.1	Wu et al. 2016^32^	LPS injection	4/group	E2	1 h before LPS	0.1 or 100 µg/Kg	30 min before, together and 30 min after	Iba-1	Striatum, hippocampus and motor cortex	na	↓
Inflammation	P2X7	Choi et al. 2007^27^	LPS animal model for inflammation	5/group	oxATP	30 min before LPS injection	1 µ	1, 4, 8, 12, 24, 48 h	OX-42	Striatum	↑	↓

*MCAo* middle cerebral artery occlusion, *SE* status epilepticus, *I/R* ischemia/reperfusion, *LPS* lypopolysaccharide, *oxATP* oxidized ATP, *BBG* brilliant blue G, *RB2* reactive blue 2, *E2* estrogen, *Iba-1* ionized calcium binding adaptor molecule 1, *OX-42* ntegrin alpha M antibody, *IB4* isolectin IB4, *na* not available, *↑* increase,*↓* decrease *------* no significant change

### Microglial ion channel antagonists in epilepsy animal models

Three studies assessed the effect of ion signaling blockade on microglial phenotypes in animal models for epilepsy. Two studies evaluated P2X7^18,19^, while only one investigated TRPC6^20^. These studies were performed on the hippocampus of corialactone and pilocarpine seizure models and showed an increase in microglial channels after induction of status epilepticus. Furthermore, all of the studies demonstrated that P2X7 and TRPC6 antagonism were related to a decrease in microglial activated state.

### Microglial ion channel antagonists in ischemic animal models

The majority of studies (4 out of 6) on ischemic models associated a decrease in microglial activation with purinergic channel blockade. Of these, three evaluated P2X7 channels^21–23^, and two of the three demonstrated a reduction in microglial activation^21,22^. Although the investigation of reactive blue 2 (RB2) in P2X7 blockade showed an increase in reactive microglia, a protective effect against the severity of post ischemic neurological impairment was seen in RB2-treated rats^23^. Studies evaluating P2X4 and P2Y12 antagonists also demonstrated a reduction in the activated state of CNS-resident immune cells^24,25^.

Regarding K^+^ channels in ischemic models, it was shown that K^+^ channel blockade by glibenclamide did not modify microglial state^26^. However, glibenclamide decreased neurological deficits and reduced neuronal death in preclinical studies^26^.

### Microglial ion channel antagonists and inflammation in animal models

One study assessed ion channel blockade and microglial cell function in an inflammatory model^27^ and demonstrated a time-dependent increase in P2X7, to a maximum level at 12 h, after injection of lipopolysaccharide (LPS). Furthermore, P2X7 antagonism with oxATP pointed to a decrease in microglial activated state^27^.

### Animal models for microglial ion channel blockade in traumatic brain injury and spinal cord injury

There was only one study evaluating P2X7 blockade and microglial activation in traumatic brain injury (TBI)^28^. According to the study, purinergic antagonism attenuated microglial activated state and improved neurobehavioral outcomes after TBI. Moreover, P2X7 blockade decreased IL-1β expression and p38 phosphorylation, increasing the survival of neurons in the injured cerebral cortex^28^.

In a study on P2X4 antagonism in microglia after spinal cord injury (SCI), Zhou et al. found that dexmedetomidine, a highly selective α2 adrenergic agonist with sedative properties, decreased microglial activation and reversed mechanical hyperalgesia^29^. In addition, spared nerve injury rats presented high levels of p38 and brain-derived neurotropic factor (BDNF) expression in the dorsal horn compared to controls, downregulated by dexmedetomidine treatment^29^.

### Microglial ion channel antagonists in pain models

One study assessed P2X7 blockade in microglial phenotype in preclinical pain models^30^. This channel expression was increased in the ipsilateral spinal cord after nerve injury. Temporal evolution in P2X7 levels in the dorsal horn of the spinal cord and the difference in P2X7 levels matched the emergence of mechanical allodynia and thermal hypersensitivity. The study showed that nerve injury-induced mechanical allodynia and thermal hypersensitivity was reversed by intrathecal administration of Brilliant Blue G (BBG)^30^.

### Microglial ion channel antagonists in animal models for Parkinson’s disease

In animal models for Parkinson’s disease, P2X7 and kir 2.1 blockade decreased microglial activation^31,32^. In rats treated with BBG, a P2X7 antagonist, p38-MAPK activation was reversed, microglial activation was attenuated, and a reduction in the loss-of-dopaminergic neurons was observed in the substantia nigra^31^. Increases in neuron survival was also demonstrated by estrogenic blockade of kir 2.1. Estrogen incremented anti-apoptotic genes and pro-survival PI3K-Akt signaling^32^.

## Discussion

Our review shows that pharmacological blockade of ionic signaling in microglia in preclinical models for epilepsy, ischemia, Parkinson, pain, TBI, SCI, and inflammation modify microglial phenotypes when compared to controls, thereby decreasing microglial activation. There is evidence of reduced microglial activation following blockade of purinergic channels, such as P2X7, P2X4, and P2Y12^18,19,24,25,27–31^. Specifically, the majority (8 out of 9) of studies investigating P2X7 channels in preclinical models for epilepsy, ischemia, TBI, SCI, Parkinson, pain, and inflammation demonstrated a decrease in microglial activated state^18,19,21,22,27,28,30,31^. However, there was an increase in microglial activation in an ischemic animal model following P2X7 blockade^23^. Variations between studies in the range of doses of ion channel antagonists and the time elapsed from the last drug treatment to microglial evaluation may explain the differences between studies. All the P2X4 and P2Y12 studies showed a decline in immune cell activation after blockade of these channels (3 out of 3 studies)^24,25,29^.

Studies with animal models for epilepsy and Parkinson’s disease showed a reduction in microglial activated state after TRPC6 and kir 2.1 blockade^20,32^. However, in an ischemic animal model, K^+^-channel antagonism with glibenclamide showed no differences in microglial state^26^. This variety in the results may occur since glibenclamide does not reach significant concentrations in the brain unless the plasma concentration is extremely high^33^. The drug binds to plasma proteins, which impairs its entry into the brain, as plasma proteins are unable to cross the blood–brain barrier^33^. Additionally, glibenclamide appears to be removed from the brain rapidly by a highly efficient efflux system when injected directly into the CNS^33^.

Taken together, these findings indicate that ion channel blockade elicits a significant decrease in microglial activated state in preclinical models for epilepsy, ischemia, TBI, SCI, inflammation, Parkinson, and pain. Nevertheless, further in vivo studies are needed to determine the effects of ion channel antagonists on microglial function (see Box 1 for suggested future directions).

### Box 1 Suggested future research directions

1. Further studies on primates to determine the effect of acute and chronic administration of ion channel antagonists on microglial function.
2. Studies comparing males and females to investigate potential gender differences.
3. Studies needed to investigate whether there are strain differences.
4. Studies to investigate the effect of acute and chronic ion channel blockade on neuronal firing and how it relates to measures of neurotransmitter release and synthesis capacity.
5. Studies to investigate correlations between microglial channels and different brain regions at different time points.
6. Studies to investigate correlations between microglial ion channel modulation by different types of drugs, such as antipsychotics, anti-epileptics, and antidepressants.
7. Development of microglia-like cells derived from human stem cells.

### General methodological considerations

Importantly, despite substantial evidence that microglial dysfunction is part of the core pathology of many neurological and psychiatric disorders, evidence remains limited for a direct causal link between a specific and druggable target in microglial cells and a specific disease. Under physiological conditions, microglia reside in the brain parenchyma, so a drug targeting microglia needs to be able to enter the CNS^1,34^. In recent years, various ion channel antagonists have been developed as potential drugs. However, most of these compounds were not designed to enter the CNS and would therefore lack the properties to be effective in CNS disorders mediated by microglial channels^34^.

Additionally, microglial cells are highly plastic. Therefore, it is not always clear whether the molecule targeted by a drug is indeed expressed by the microglia^34^. For example, various P2 receptors have been suggested as drug targets in microglia. However, the expression pattern of P2 receptors strongly depends on the cell phenotype^35^. For example, microglia may lose P2Y12 receptors in the disease state, whereas P2X4 and P2X7 expression is often increased in microglia in disease^35–37^. Thus, the spatial-temporal effect of microglial channel antagonists still needs to be tested directly (Box 1).

General limitations of the selected studies were that the strain- and sex-specific effects of acute and repeated ion channel blockade on microglial function have still not been tested directly (Box 1). There is evidence for sexual dimorphism in microglial function and a sex bias in CNS disorders with microglial pathology^37^.

In an attempt to produce more homogeneous outcomes and better understand how pharmacological blockade of microglial ion channels alters microglial state, our search strategy only included studies that evaluated microglia in the activated state (ameboid morphology) or resting state (ramified morphology) as evidenced by microglial immunohistochemical analysis. Moreover, studies that evaluated only in vitro ion channel blockade were also excluded. This is justified due to the highly reactive nature of microglia, which substantially alters their morphology and functional properties when exposed to culture conditions^15^. The two restrictions significantly reduced the number of studies included in this review.

Our review also included different types of microglial ion channels. Although we found that ion channel blockade was associated with a decrease in microglial activated state in 13 of 15 studies, caution is necessary when interpreting this result. Importantly, microglia have different types of ion channels that play a variety of roles^1^. It is thus plausible that some ion channel antagonists decrease microglial function while others increase it. Our search criteria yielded studies showing that P2X7, P2X4, P2Y12, kir 2.1, and TRPC6 blockade were related to a decrease in microglial activated state. However, future studies should be performed on different types of ion channels and their association with microglial phenotypes. Furthermore, although our review demonstrated that ionic signaling blockade decreases microglial activation, the majority of studies were conducted in rodent models. Thus, extrapolation of microglial ion channel modulation to primates should be treated with caution, and further studies are needed (Box 1).

### Hypothesis on the effects of ion channel antagonists on microglial function

**Fig. 2:**
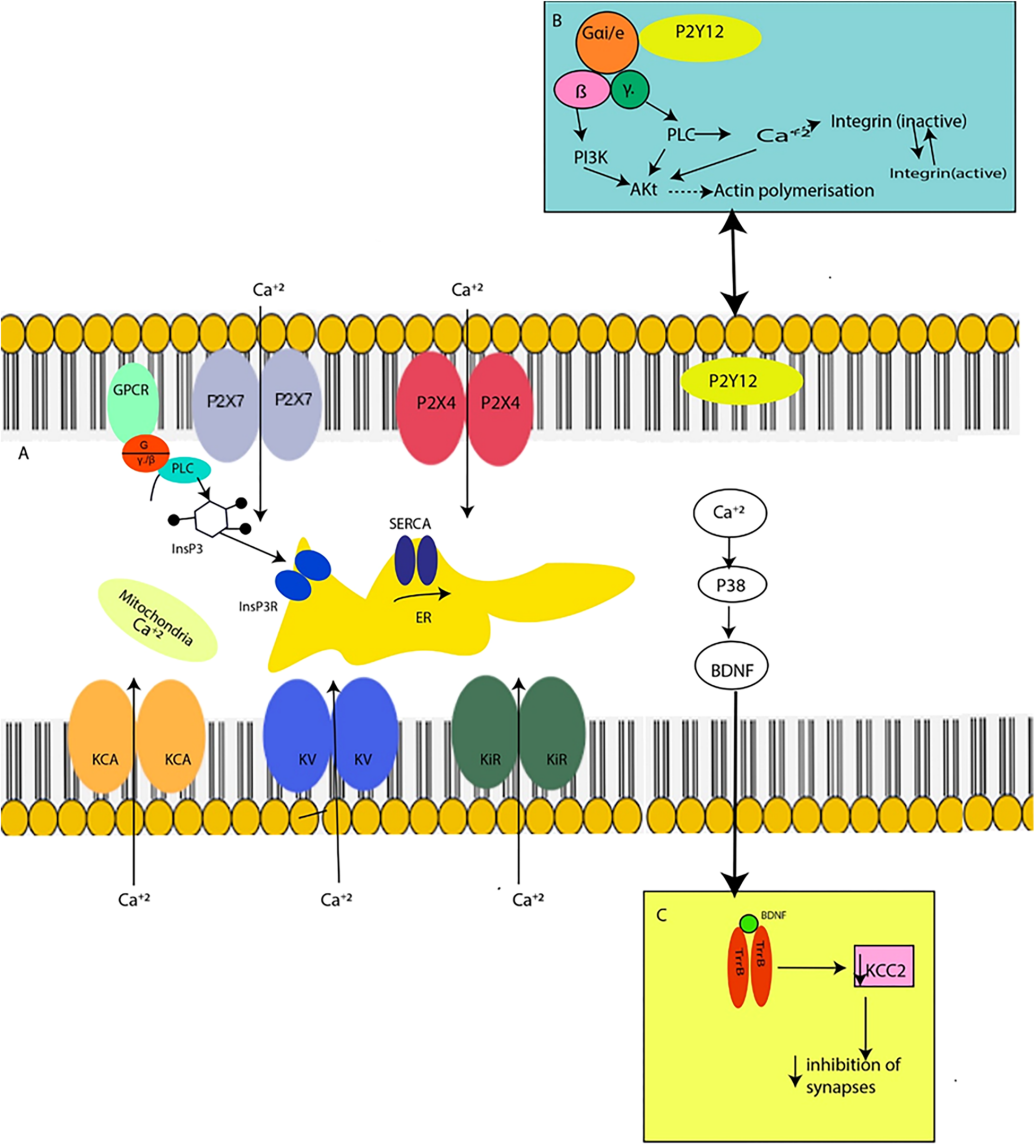
Ca^+2^ signaling through microglial ion channels. **a** Extrusion of Ca^+2^ from the cytosol is accomplished by Ca^+2^ uptake into the ER via SERCA and Ca^+2^ accumulation in mitochondria. A significant Ca^+2^ influx and downstream activation of Ca^+2^-dependent intracellular pathways, among which the phosphorylation of p38 MAP kinase or the activation of the PI3K/Akt pathway, are directly involved in microglial functions such as synthesis and release of BDNF and chemotactic responses. **b** Activation of PI3K and PLC accompanied by a rise in intracellular Ca^+2^, subsequently triggers phosphorylation and hence activation of Akt. This results in the enhanced expression and activation of integrins, promoting adhesion of microglial processes to the extracellular matrix, causing reorganization of the actin cytoskeleton and leading to chemotaxis. **c** BDNF rapidly downregulates KCC2 through TrkB, causing a deficiency in synaptic inhibition

The underlying mechanism of ion channel action in microglia remains to be fully established. However, several lines of evidence indicate that the mechanism involves Ca^+2^ signaling^38–40^. Microglial activation occurs via a cascade of extracellular and intracellular signaling events beginning with cell surface receptor and ligand-gated ion channel activation followed by short-term and long-term changes in intracellular Ca^+2^^40^. These Ca^+2^ signals can cause release of other factors in autocrine-paracrine feedback loops and are necessary for various subsequent cellular responses in microglia^39,40^ (Fig. 2).

Microglia contain at least two types of intracellular Ca^+2^ stores: the mitochondria and the endoplasmic reticulum (ER)^1^. The main pathway for generation of intracellular Ca^+2^ signaling is associated with inositol 1,4,5-trisphosphate (InsP3) receptors on the ER membrane^1,40^. Stimulation of G protein-coupled metabotropic receptors results in the activation of phospholipase C (PLC), production of two secondary messengers, including diacylglycerol and InsP3, and Ca^+2^ release from the ER^1^. Importantly, ER depletion activates store-operated Ca^+2^ entry (SOCE), known as a capacitive Ca^+2^ influx, mediated by plasmalemmal channels such as calcium release-activated Ca^+2^ (CRAC) channels and/or transient receptor potential (TRP) channels^1^. In addition, STIM1, one of the ER membrane proteins, senses the filling state of ER Ca^+2^ and delivers the ER to the plasma membrane, where it directly activates Orai1/CRAC channels, thereby facilitating the reuptake of Ca^+2^ to ER through the endoplasmic reticulum Ca^+2^-ATPases (SERCA). Ca^+2^ concentration in the ER is precisely controlled by SERCA^1^. The influx of Ca^+2^ through ion channels plays an important role in many inflammatory processes, including microglial activation^40^.

In response to an injury, various extracellular signals may activate microglia and upregulate ion channels. For instance, proinflammatory cytokines upregulate kir, TRPC6, and KCa expression, which may cause hyperpolarization of microglial cells^41^. Additionally, not only cytokines but also ATP can cause an increase in purinergic receptors, which are highly Ca^+2^ permeable^1^. A significant Ca^+2^ influx and the downstream activation of Ca^+2^-dependent intracellular pathways, among which the phosphorylation of p38 MAP kinase or the activation of the PI3K/Akt pathway, are directly involved in microglial functions such as synthesis and release of BDNF and chemotactic responses^1^.

Under pathological conditions, one of the first activities at the injury site is ATP release, activating microglia^40^. ATP binds to P2X7, a “sensor of danger”, driving resting microglial cells into the activated form, forming large pores and allowing Ca^+2^ entry^40^. Ca^+2^ entry is also increased by the inward rectifier K currents (ikir), an early marker for activated microglia^4^. Furthermore, extracellular ATP induces membrane ruffling and microglial chemotaxis mediated by the Gi/o protein-coupled P2Y12 receptor^1^. P2Y12 receptor-mediated activation of the PI3K pathway and increased Akt phosphorylation are required for microglial chemotaxis in response to ATP^1^.

Microglia exert phagocytic activity at the site of injury. Microglial phagocytosis can initiate respiratory burst, a key function of activated microglia that produces toxic reactive oxygen species^42^. The K^+^-channels are involved in controlling microglial respiratory burst^42^, whereas P2X7 channels are associated with NADPH-oxidase and consequently with oxidative stress^1^. Phagocytic activity of microglial cells is felt to be involved in synapse removal during development and potentially in pruning synapses in the postnatal brain, which is associated with different psychiatric and neurological disorders^9,11^. Microglial phagocytosis is intimately associated with cytokine modulation and may contribute to synaptic plasticity^43^.

### Microglial action on synaptic plasticity

Microglia may regulate synaptic plasticity by modulating Cl^−^ gradient in neurons through microglial BDNF release and purinergic signaling^44^. The purinergic receptors P2X7, P2Y12, and P2X4 are related to p38 MAP kinase, which is directly involved in BDNF synthesis and release^45^. BDNF release occurs through Ca^+2^-regulated exocytosis, and regulation of BDNF synthesis involves the p38-MAPK signaling pathway^46^. BDNF rapidly downregulates K^+^- Cl^−^ cotransporter 2 (KCC2) through receptor tyrosine kinase B (TrkB)^46^. KCC2 activity maintains a low intracellular Cl^−^ concentration, a prerequisite for effective GABA/Gly-mediated inhibition in the nervous system^47^. Thus, since microglial BDNF release decreases KCC2 activity, there is a deficiency in the inhibition of synapses, increasing neuronal hyperexcitability. However, microglial channels could also be linked to neuronal hyperexcitability in a different way: BDNF secreted following stimulation of microglial purinergic receptors may induce phosphorylation of NR1 subunit of NMDA neuronal receptors. This may contribute to neuronal hyperexcitability^48^.

### Implications for neurological and psychiatric disorders

Microglial activation is related to a variety of neurological and psychiatric disorders^49^. This activated state is related to an excitatory-inhibitory imbalance, generating oxidative stress^50^, modifying BDNF release^51^, and changing molecular signaling^52,53^. Additionally, in the brain, Cl^−^ homeostasis and the reduction of KCC2 activity has been associated with several neurological and psychiatric disorders, such as epilepsy, neuropathic pain, autism spectrum disorders, affective disorders, and schizophrenia^54–57^. Several lines of evidence accumulated during the last decade have indeed demonstrated that the increase in excitability in these pathological conditions can be largely explained by a loss of inhibition, and KCC2 has been recognized as an important molecular target underlying this loss^55,56^.

It is thus plausible that Ca^+2^ entry through microglial ion channels may activate microglia and influence MAPK and AKT pathways. This could alter microglial phagocytosis functions, modify BDNF release, and increase neuronal hyperexcitability through KCC2 inhibition, contributing to the pathogenesis of mental disorders. Furthermore, first-line treatments for neurological and psychiatric disorders have potential inhibitory effects on microglia. To name a few, antipsychotics such as haloperidol, risperidone, olanzapine, clozapine, and chlorpromazine inhibit microglial proton currents, suppressing reactive oxygen production, acting as anti-inflammatory modulators^58^. Proton currents are also inhibited by the SSRIs paroxetine and sertraline, for example, attenuating the mobilization of intracellular Ca^+2^ ^59^. Aripiprazole also downregulates microglial Ca^+2^ signaling^59^, while carbamazepine suppresses microglial activation via p38-MAPK. Additionally, these drugs interfere with neurotransmitters, such as dopamine, serotonin, acetylcholine, and norepinephrine. This could contribute to further alterations in microglial phenotypes. Norepinephrine inhibits microglial inflammatory reactions through activation of cyclic AMP and suppression of downstream MAPK^60^. Dopamine and serotonin could interfere with microglial chemotaxis, and acetylcholine may alter Ca^+2^ signaling^60^. These effects could additionally contribute to a decrease in microglial activated state in neurological and psychiatric disorders.

Calcium-channel blockers might also contribute to a decrease in microglial activated state^61,62^, providing potential utility in the treatment of neurological and psychiatric disorders^63–65^. In fact, calcium-channel blockers have been used successfully to treat absence seizures^63^, and are emerging as potential therapeutic targets for pathologies, such as Parkinson disease, pain, mood disorders, and anxiety^63^. For instance, centrally acting calcium-channel blockers targeting Ca^2+^ channels of dopaminergic neurons might decrease risk of Parkinson disease^66^, the calcium-channel blocker isradipine could be efficacious for bipolar disorder^65^, and the drug nifedipine might have an antiepileptic effect^67^. Nevertheless, clinical trials with calcium-channel blockers in neurological and psychiatric disorders present inconsistent results^64^. One possible reason for this is the pharmacokinetic variability among different calcium-channel blockers^68^. Dihydripyridines, such as isradipine, have more favorable brain–blood barrier penetration and binding to calcium-channel compared to other calcium-channel blockers, such as diltiazem and verapamil^68^. Therefore, although there is evidence supporting the utility of calcium channel blockers in a variety of psychiatric and neurological disorders, what remains to be understood is how to develop new types of calcium channel inhibitors that specifically target calcium channels that are involved in pathophysiological processes, while sparing those that contribute to normal physiological function^63^. This requires an extensively understanding of how calcium channels participate in the function of specific brain circuits that are implicated in pathophysiology and how these channels may be dysregulated in pathological states^63^. This represents an enormous challenge in finding compounds that effectively cross the blood–brain barrier, have high affinity and target selectivity^63^.

Clinical trials with drugs targeting-specific ion channels are currently underway. Some P2X7 receptor antagonists have been developed as potential drugs. For example, AZD9056^69^ and CE-224,535^70^ have been tested in clinical trials in rheumatoid arthritis, while GSK1482160^71^ was developed to target peripheral pain. These compounds were not designed to enter the CNS and would therefore most likely lack the properties to be effective in nervous system disorders that could be associated with microglial channels. Nevertheless, as our understanding of the role of ion channels on microglia in human diseases grows, the development of therapeutics targeting-specific microglial molecules will probably occur. However, some challenges remain for the development of microglial channel antagonists for use in humans, such as target specificity, CNS penetrance, and differences between animal and human microglia. New research strategies such as the development of microglia-like cells derived from human stem cells might help overcome the current limitations (Box 1).

## Conclusion

The administration of ion channel antagonists leads to a decrease in microglial activation in different preclinical models. These findings suggest that ion channels have convergent pathways that potentially contribute to differentiation of microglial phenotypes and functions, which could be altered in a variety of neurological and psychiatric disorders. Further studies are required to explore whether the same antagonists’ effects are seen in primate brain microglia and to investigate-specific druggable targets in microglial cells.

## Electronic supplementary material

Supplementary Material

Supplementary Material 7

## References

[1] Kettenmann H, Hanisch UK, Noda M, Verkhratsky A (2011). Physiology of microglia. Physiol. Rev..

[2] Nimmerjahn A, Kirchhoff F, Helmchen F (2005). Resting microglial cells are highly dynamic surveillants of brain parenchyma in vivo. Science.

[3] Schilling T, Eder C (2007). Ion channel expression in resting and activated microglia of hippocampal slices from juvenile mice. Brain Res..

[4] Nguyen HM (2017). Differential Kv1.3, KCa3.1, and Kir2.1 expression in “classically” and “alternatively” activated microglia. Glia.

[5] O’Hare Doig RL (2017). Specific ion channels contribute to key elements of pathology during secondary degeneration following neurotrauma. BMC Neurosci..

[6] Gueguinou M (2014). KCa and Ca(2+) channels: the complex thought. Biochim. Biophys. Acta.

[7] Liu N (2017). NF-kappaB dependent up-regulation of TRPC6 by Abeta in BV-2 microglia cells increases COX-2 expression and contributes to hippocampus neuron damage. Neurosci. Lett..

[8] Burnstock G (2016). An introduction to the roles of purinergic signalling in neurodegeneration, neuroprotection and neuroregeneration. Neuropharmacology.

[9] Schafer DP (2012). Microglia sculpt postnatal neural circuits in an activity and complement-dependent manner. Neuron.

[10] Tremblay ME, Lowery RL, Majewska AK (2010). Microglial interactions with synapses are modulated by visual experience. PLoS Biol..

[11] Stevens B (2007). The classical complement cascade mediates CNS synapse elimination. Cell.

[12] Zhang B, Zou J, Han L, Rensing N, Wong M (2016). Microglial activation during epileptogenesis in a mouse model of tuberous sclerosis complex. Epilepsia.

[13] Zhan Y (2014). Deficient neuron-microglia signaling results in impaired functional brain connectivity and social behavior. Nat. Neurosci..

[14] Villegas-Llerena C, Phillips A, Garcia-Reitboeck P, Hardy J, Pocock JM (2016). Microglial genes regulating neuroinflammation in the progression of Alzheimer’s disease. Curr. Opin. Neurobiol..

[15] Hellwig S, Heinrich A, Biber K (2013). The brain’s best friend: microglial neurotoxicity revisited. Front. Cell Neurosci..

[16] Moher D, Liberati A, Tetzlaff J, Altman DG (2009). Preferred reporting items for systematic reviews and meta-analyses: the PRISMA statement. PLoS Med..

[17] Hooijmans CR (2014). SYRCLE’s risk of bias tool for animal studies. BMC Med. Res. Methodol..

[18] Choi HK (2012). The roles of P2X7 receptor in regional-specific microglial responses in the rat brain following status epilepticus. Neurol. Sci..

[19] Huang C (2017). Inhibition of P2X7 receptor ameliorates nuclear factor-Kappa B mediated neuroinflammation induced by status epilepticus in rat hippocampus. J. Mol. Neurosci..

[20] Lee SK, Kim JE, Kim YJ, Kim MJ, Kang TC (2014). Hyperforin attenuates microglia activation and inhibits p65-Ser276 NFkappaB phosphorylation in the rat piriform cortex following status epilepticus. Neurosci. Res..

[21] Yu Q (2013). Block of P2X7 receptors could partly reverse the delayed neuronal death in area CA1 of the hippocampus after transient global cerebral ischemia. Purinergic. Signal..

[22] Chu K (2012). Inhibition of P2X7 receptor ameliorates transient global cerebral ischemia/reperfusion injury via modulating inflammatory responses in the rat hippocampus. J. Neuroinflamm..

[23] Melani A (2006). P2X7 receptor modulation on microglial cells and reduction of brain infarct caused by middle cerebral artery occlusion in rat. J. Cereb. Blood Flow Metab..

[24] Wixey JA, Reinebrant HE, Carty ML, Buller KM (2009). Delayed P2X4R expression after hypoxia-ischemia is associated with microglia in the immature rat brain. J. Neuroimmunol..

[25] Gelosa P (2014). Microglia is a key player in the reduction of stroke damage promoted by the new antithrombotic agent ticagrelor. J. Cereb. Blood Flow Metab..

[26] Ortega FJ (2012). ATP-dependent potassium channel blockade strengthens microglial neuroprotection after hypoxia-ischemia in rats. Exp. Neurol..

[27] Choi HB, Ryu JK, Kim SU, McLarnon JG (2007). Modulation of the purinergic P2X7 receptor attenuates lipopolysaccharide-mediated microglial activation and neuronal damage in inflamed brain. J. Neurosci..

[28] Liu X (2017). Inhibition of P2X7 receptors improves outcomes after traumatic brain injury in rats. Purinergic Signal..

[29] Zhou TT, Wu JR, Chen ZY, Liu ZX, Miao B (2014). Effects of dexmedetomidine on P2X4Rs, p38-MAPK and BDNF in spinal microglia in rats with spared nerve injury. Brain Res..

[30] He WJ (2012). Spinal P2X(7) receptor mediates microglia activation-induced neuropathic pain in the sciatic nerve injury rat model. Behav. Brain Res..

[31] Wang XH, Xie X, Luo XG, Shang H, He ZY (2017). Inhibiting purinergic P2X7 receptors with the antagonist brilliant blue G is neuroprotective in an intranigral lipopolysaccharide animal model of Parkinson’s disease. Mol. Med. Rep..

[32] Wu SY (2016). Estrogen ameliorates microglial activation by inhibiting the Kir2.1 inward-rectifier K(+) channel. Sci. Rep..

[33] Lahmann C, Kramer HB, Ashcroft FM (2015). Systemic administration of glibenclamide fails to achieve therapeutic levels in the brain and cerebrospinal fluid of rodents. PLoS ONE.

[34] Biber K, Moller T, Boddeke E, Prinz M (2016). Central nervous system myeloid cells as drug targets: current status and translational challenges. Nat. Rev. Drug Discov..

[35] Koizumi S, Ohsawa K, Inoue K, Kohsaka S (2013). Purinergic receptors in microglia: functional modal shifts of microglia mediated by P2 and P1 receptors. Glia.

[36] Inoue K (2008). Purinergic systems in microglia. CMLS.

[37] Crain JM, Nikodemova M, Watters JJ (2009). Expression of P2 nucleotide receptors varies with age and sex in murine brain microglia. J. Neuroinflamm..

[38] Ikeda M (2013). Ca(2+) spiking activity caused by the activation of store-operated Ca(2+) channels mediates TNF-alpha release from microglial cells under chronic purinergic stimulation. Biochim. Biophys. Acta.

[39] Korvers L (2016). Spontaneous Ca(2+) transients in mouse microglia. Cell Calcium.

[40] Farber K, Kettenmann H (2006). Functional role of calcium signals for microglial function. Glia.

[41] Deftu AF, Ristoiu V, Suter MR (2018). Intrathecal administration of CXCL1 enhances potassium currents in microglial cells. Pharmacology.

[42] Khanna R, Roy L, Zhu X, Schlichter LC (2001). K+channels and the microglial respiratory burst. Am. J. Physiol. Cell Physiol..

[43] Riazi K (2008). Microglial activation and TNFalpha production mediate altered CNS excitability following peripheral inflammation. Proc. Natl Acad. Sci. USA.

[44] Coull JA (2005). BDNF from microglia causes the shift in neuronal anion gradient underlying neuropathic pain. Nature.

[45] Trang T, Beggs S, Wan X, Salter MW (2009). P2X4-receptor-mediated synthesis and release of brain-derived neurotrophic factor in microglia is dependent on calcium and p38-mitogen-activated protein kinase activation. J. Neurosci..

[46] Rivera C (2002). BDNF-induced TrkB activation down-regulates the K(+)–Cl(−) cotransporter KCC2 and impairs neuronal Cl(−) extrusion. J. Cell Biol..

[47] Rivera C (1999). The K+/Cl- co-transporter KCC2 renders GABA hyperpolarizing during neuronal maturation. Nature.

[48] Caldeira MV (2007). BDNF regulates the expression and traffic of NMDA receptors in cultured hippocampal neurons. Mol. Cell Neurosci..

[49] Salter MW, Stevens B (2017). Microglia emerge as central players in brain disease. Nat. Med..

[50] Hassan W (2016). Association of oxidative stress with psychiatric disorders. Curr. Pharm. Des..

[51] Autry AE, Monteggia LM (2012). Brain-derived neurotrophic factor and neuropsychiatric disorders. Pharmacol. Rev..

[52] Naziroglu M, Demirdas A (2015). Psychiatric disorders and TRP channels: focus on psychotropic drugs. Curr. Neuropharmacol..

[53] Chen HM (2014). Transcripts involved in calcium signaling and telencephalic neuronal fate are altered in induced pluripotent stem cells from bipolar disorder patients. Transl. Psychiatry.

[54] Tao R (2012). Transcript-specific associations of SLC12A5 (KCC2) in human prefrontal cortex with development, schizophrenia, and affective disorders. J. Neurosci..

[55] Di Cristo G, Awad PN, Hamidi S, Avoli M (2018). KCC2, epileptiform synchronization, and epileptic disorders. Prog. Neurobiol..

[56] Ford A (2015). Engagement of the GABA to KCC2 signaling pathway contributes to the analgesic effects of A3AR agonists in neuropathic pain. J. Neurosci..

[57] Corradini I (2018). Maternal immune activation delays excitatory-to-inhibitory gamma-aminobutyric acid switch in offspring. Biol. Psychiatry.

[58] Shin H, Kim J, Song JH (2015). Clozapine and olanzapine inhibit proton currents in BV2 microglial cells. Eur. J. Pharmacol..

[59] Horikawa H (2010). Inhibitory effects of SSRIs on IFN-γ induced microglial activation through the regulation of intracellular calcium. Prog. Neuropsychopharmacol. Biol. Psychiatry.

[60] Kato TA (2013). Neurotransmitters, psychotropic drugs and microglia: clinical implications for psychiatry. Curr. Med. Chem..

[61] Huang BR (2014). Anti-neuroinflammatory effects of the calcium channel blocker nicardipine on microglial cells: implications for neuroprotection. PLoS ONE.

[62] Hashioka S, Klegeris A, McGeer PL (2012). Inhibition of human astrocyte and microglia neurotoxicity by calcium channel blockers. Neuropharmacology.

[63] Zamponi GW (2016). Targeting voltage-gated calcium channels in neurological and psychiatric diseases. Nat. Rev. Drug Discov..

[64] Cipriani A (2016). A systematic review of calcium channel antagonists in bipolar disorder and some considerations for their future development. Mol. Psychiatry.

[65] Ostacher MJ (2014). Pilot investigation of isradipine in the treatment of bipolar depression motivated by genome-wide association. Bipolar Disord..

[66] Ritz B (2010). L-type calcium channel blockers and Parkinson’s disease in Denmark. Ann. Neurol..

[67] Damasceno D, Ferreira A, Doretto M, Almeida A (2012). Anticonvulsant and antiarrhythmic effects of nifedipine in rats prone to audiogenic seizures. Braz. J. Med. Biol. Res..

[68] Casamassima F (2010). L-type calcium channels and psychiatric disorders: a brief review. AJMG.

[69] Keystone EC, Wang MM, Layton M, Hollis S, McInnes IB (2012). Clinical evaluation of the efficacy of the P2X7 purinergic receptor antagonist AZD9056 on the signs and symptoms of rheumatoid arthritis in patients with active disease despite treatment with methotrexate or sulphasalazine. Rheum. Dis..

[70] Stock TC (2012). Efficacy and safety of CE-224,535, an antagonist of P2X7 receptor, in treatment of patients with rheumatoid arthritis inadequately controlled by methotrexate. J. Rheumatol..

[71] Ali Z (2013). Pharmacokinetic and pharmacodynamic profiling of a P2X7 receptor allosteric modulator GSK1482160 in healthy human subjects. Br. J. Clin. Pharmacol..

