# Supplementary material for: The role of convergent ion channel pathways in microglial phenotypes: a systematic review of the implications for neurological and psychiatric disorders
Source: Transl Psychiatry. 2018 Nov 29;8:259. doi: 10.1038/s41398-018-0318-0 (PMC6265266; doi:10.1038/s41398-018-0318-0)
Supplement: Supplementary file 1 — Supplementary Material [file 41398_2018_318_MOESM1_ESM.docx]

**Appendix 1: Search Protocol**

# Systematic Search Protocol

*Written based on the WHO Review Protocol Template, 2011*

Title: **The role of ion channels convergent pathways on microglial phenotypes: a systematic review of the implications for neurological and psychiatry disorders.**

1. Background: Microglia activation and ion signaling are linked characteristic of neuropsychiatric disorders. However, it is not clear specifically the molecular mechanisms that exist between these conditions.
2. Objective: This review aims to bring together all the research on how ionic channels interacts with microglia cells to identify a pattern of immune response activation.

1. Review Question (Population Intervention Comparison Outcome)

Population: microglia cells

Intervention: activation and /or inhibition of ion channels

Comparison: without activation and /or inhibition of ion channels

Outcome: microglia cells active or at resting stage

Evidence Gathering and Study Selection: Web of Science and Pubmed databases will be searched. Full search terms for each database and results found per database are listed in the Appendix 2.

Reference searches: Bibliographies of papers deemed eligible for this review will be hand searched to identify any additional eligible references, which will then be screened for title, abstract or full text as appropriate.

1. Eligibility Criteria

The results of these searches will be combined and deduped using Endnote. They will then be screened for title and abstract, and then full text using the following eligibility criteria.

1. Type of study included: any type of study design.
2. Types of participants: Studies that examine the relationship between ion channels and microglia will be included
3. Types of outcome measures: microglia in an activate or at resting state.

1. Exclusion Criteria

Reviews, studies which lacked a baseline condition or control group, studies that did not report original data, studies without an immunohystochemical analyses of microglia, studies evaluating genetic deficiency of ionic channels, in vitro studies will be excluded.

1. Data extraction

Data extracted will include:

- - Citation information
  - Subject age, sex and species, duration in fixative solution, brain region examined, method of measuring microglia state used and results. The final decision on what to include in the published tables will be made by the systematic review author team based on importance and variability within the studies.

1. Data Synthesis

Narrative synthesis is planned, with the same disorders/conditions grouped together as appropriate depending on number of papers and commonalities in measurement. Statistical synthesis is not expected to be possible as there is wide variability in the types of measures, types of outcomes and brain regions investigated, but will be considered if feasible given the data.

1. Dissemination

A manuscript will be prepared for submission to a peer reviewed journal in the neuroscience field.

|  | \|  \|  \| \| --- \| --- \|   **Appendix 2: Search terms and results** | | | | | | | |  |
| --- | --- | --- | --- | --- | --- | --- | --- | --- | --- | --- | --- |
| #18 | #1 AND #16 AND #17 | | | | | | | |  |
| #17 | #11 OR #10 OR #9 OR #8 OR #7 OR #6 OR #5 OR #4 OR #3 OR #2 | | | | | | | |  |
| #16 | #15 OR #14 OR #13 OR #12 | | | | | | | |  |
| #15 | (gyrus orbital or sulcus olfactoryor convolutions superior frontal or orbitofrontal cortices lateral or gyrus frontalis superior or rectal gyrusor cortices ventromedial prefrontal or orbital cortices or cortex orbital or prefrontal cortices ventromedial or inferiors gyrus frontalis or orbital gyri or orbital area or convolution superior frontal or frontalis superiors gyrus or inferior frontal gyrus or gyri orbitofrontal or orbitofrontal regions or frontalis inferiors gyrus or frontal sulcus or prefrontal cortex ventromedial or straight gyrus or cortex lateral orbitofrontal or gyrus frontalis inferior or sulciolfactory or orbital areas or orbitofrontal gyri or area orbital or orbitofrontal region or cortices lateral orbitofrontal or lateral orbitofrontal cortex or superior frontal convolution or cortex orbitofrontal or medial frontal gyrus or gyrus orbitofrontal or gyrus straight or superior frontal gyrus or frontal gyrus medial or ventromedial prefrontal cortex or gyrus rectal or subcallosal area or olfactory sulcus or prefrontal cortex or superior frontal convolutions or sulcus frontal or olfactory sulci or region orbitofrontal or superiors gyrus frontalis or superior gyrus frontalis or gyrus superior frontal ororbitofrontal cortex or frontal gyrus inferior or gyrus frontalis inferiors or cortex ventromedial prefrontal or marginal gyrus or rectus gyrus or orbital cortex or gyrus medial frontal or orbitali gyrus or orbitofrontal gyrus or inferior gyrus frontalis or frontal gyrus superior or gyri orbital or areas orbital or cortex prefrontal or cortices orbital or gyrus rectus or frontalis superior gyrus or lateral orbitofrontal cortices or orbitofrontal cortices or gyrus frontalis superiors or orbitofrontal cortex lateral or gyrus marginal or Broca or Neocortex or neocortical molecular layer or neocortices cerebral or isocortex or cerebral neocortices or neopalliumsor corticalis substantiaor multiforme layer neocortical or neocortical multiform layer or layer neocortical molecular or cortices neopallial or neopallial cortex or neocortical internal pyramidal layer or molecular layer neocortical or neopallial corticesor cortex neopallial or layers neocortical multiform or neocortex cerebralor molecular layers neocortical or neocortical internal granular layer or neocortical multiform layers or cerebral neocortex or neocortical external pyramidal layer or neocortical molecular layers or isocortices or external granular layer or substantia corticali or corticali substantia or layer neocortical multiformor multiform layers neocortical or Occipital Lobe or occipital cortex or cuneus or gyrus annectant or sulcus lunate or gyrus lingual or gyrus occipitalor calcarine fissures or regions occipital or occipitotemporal gyrus medial or sulcus calcarine or lunate sulcus or occipital lobe or gyrus medial occipitotemporal or cuneate lobule or region occipital or fissures calcarine or calcarinus sulcus or sulcus calcarinus or lobe occipital or lobes occipital or occipital region or gyrus lingualis or occipital gyrus or annectant gyrus or occipital regions or lobules cuneate or occipital sulcus or cortices occipital or calcarine sulcus or fissure calcarine  or lingual gyrus or lobule cuneate or cortex cuneus or linguali gyrus or Visual Cortex or primary visual cortices or visual cortex primaries or primaries visual cortex or cortices extrastriate or visual cortices primary or extrastriate cortices or cortex primaries visual or cortex primary visual or striate cortex or cortices primary visual or visual cortex primary or visual cortex or cortex striate or cortex extrastriate or cortex visual or extrastriate cortex or primary visual cortex or Olfactory Cortex or Basal Forebrain or Piriform Cortex or sulcus intraparietal or regions parietal or lobes parietal or paracentral lobules posterior or gyrus supramarginal or precuneus cortices or parietal cortex or gyrus angulari or praecuneus or gyrus angularis or gyrus supramarginali or parietal regions or gyrus prelunate or lobules parietal or lobe parietal or parietal cortices posterior or gyrus supramarginals or angulari gyrus or supramarginali gyrus or marginal sulcus or posterior parietal cortex or prelunate gyrus or posterior parietal cortices or intraparietal sulcus or angularis gyrus or region parietal or parietal lobules or precuneus or cortex parietal or gyrus angular or precuneus cortex or lobule parietal or parietal lobule or cortices precuneus or posterior paracentral lobule or lobules posterior paracentral or sulcus marginal or posterior paracentral lobules or Parietal Lobe or sulcus intraparietal or regions parietal or lobes parietal or paracentral lobules posterior or gyrus supramarginal or precuneus  cortices or parietal cortex or gyrus angulari or praecuneus or gyrus angularis or gyrus supramarginali or parietal regions or gyrus prelunate or lobules parietal or lobe parietal or parietal cortices posterior or gyrus supramarginalis or angulari gyrus or supramarginali gyrus or marginal sulcus or posterior parietal cortex or prelunate gyrus or posterior parietal cortices or intraparietal sulcus or angularis gyrus or region parietal or parietal lobules or precuneus or cortex parietal or gyrus angular or precuneus cortex or lobule parietal or parietal lobule or cortices precuneus or posterior paracentral lobule or lobules posterior paracentral or sulcus marginal or posterior paracentral lobules or omatosensory Cortex or postcentral gyrus or somatosensory cortices primary or somatosensory cortex primary or cortex anterior parietal or cortices anterior parietal or cortices primary somatosensory or anterior parietal cortices or cortex secondary sensory or areas primary somatosensory or primary somatosensory cortices or cortex si or gyrus post central or secondary somatosensory areas or post central gyrus or parietal cortices anterior or gyrus postcentrali or secondary somatosensory cortex or somatosensory cortex or areas secondary somatosensory or somatosensory cortex secondary or area primary somatosensory or postcentralis gyrus or cortices secondary sensory or secondary sensory cortex or primary somatosensory cortex or somatosensory areas secondary or si cortex or primary somatosensory areas or secondary somatosensory cortices or gyrus postcentralis or area secondary somatosensory or primary somatosensory area or cortex primary somatosensory or secondary somatosensory area or secondary sensory cortices or cortices secondary somatosensory or primary somatic sensory area or postcentrali gyrus or gyrus postcentral or Wernicke Area or Sensorimotor Cortex or Auditory Cortex or gyrus transverse temporal or auditory areas temporal or temporal auditory areas or areas auditory or cortex primary auditory or transverse temporal gyrus or cortex auditory or auditory areas or transverse temporal gyri or convolutions heschl's or auditory cortex or auditory area or auditory córtex primary or auditory cortices primary or areas temporal auditory or area auditory or gyri transverse temporal or primary auditory cortices or heschl gyri or heschl convolutions or temporal gyri transverse or auditory area temporal or temporal auditory area or heschls gyri or primary auditory cortex or Temporal Lobe or temporal operculums or superior temporal gyrus or occipito-temporal gyrus lateral or gyrus fusiform or gyrus lateral occipito-temporal or gyrus temporalis superior or occipitotemporal gyrus or horns temporal or inferior horn of lateral ventricle or temporal sulcus or regions temporal or operculums temporal or cortex temporal or lobes temporal or planum polares or temporalis superior gyrus or gyrus superior temporal or horn temporal or lobe temporal or temporal horn or temporal cortices or gyrus lateral occipitotemporal or temporalis superiors gyrus or region temporal or fusiformi gyrus or temporal region or temporal horns or lateral occipito-temporal gyrus or temporal cortex or gyrus temporal or temporal operculum or temporal regions or operculum temporal or cortices temporal or temporal horn of the lateral ventricle or polare planum or fusiformis gyrus or sulcus temporal or polares planum or planum polare or temporal lobe or Diagonal Band of Broca or diagonal band of broca or broca diagonal band or External Capsule or olfactory tracts or olfactory tract lateral or main olfactory bulbs or bulbs main olfactory or bulb olfactory or glomerulus olfactory or lateral olfactory tracts or olfactory tract or bulb main olfactory or olfactory bulbs or olfactory bulb main or accessory olfactory bulb or accessory olfactory bulbs or tracts olfactory or bulbs accessory olfactory or olfactory glomerulus or bulbs olfactory or olfactory bulb accessory or tract olfactory or bulb accessory olfactory or tract lateral olfactory or olfactorius bulbus or olfactory bulb or lateral olfactory tract or Olfactory Bulb or olfactory tracts or olfactory tract lateral or main olfactory bulbs or bulbs main olfactory or bulb olfactory or glomerulus olfactory or lateral olfactory tracts or olfactory tract or bulb main olfactory or olfactory bulbs or olfactory bulb main or accessory olfactory bulb or accessory olfactory bulbs or tracts olfactory or bulbs accessory olfactory or olfactory glomerulus or bulbs olfactory or olfactory bulb accessory or tract olfactory or bulb accessory olfactory or tract lateral olfactory or olfactorius bulbus or olfactory bulb or lateral olfactory tract or Telencephalic Commissures or Anterior Cerebellar Commissure or Corpus Callosum or corpus callosums or commissures neocortical or neocortical commissures or corpus callosum or callosums corpus or interhemispheric commissure or interhemispheric commissures or neocortical commissure or callosum corpus or commissures interhemispheric or commissure interhemispheric or commissure neocortical or Internal Capsule or internal capsules or interna capsula or capsules internal or capsule internal or capsula internas or  internal capsule or capsula interna or internas capsula or Myelencephalon) |  | | | | | | |  |
| #14 | (anterior pulvinar nucleus or pulvinar nucleus or nucleus anterior pulvinar or lateral pulvinar nucleus or pulvinar nucleus oral or oral pulvinar nucleus or pulvinar nucleus inferior or pulvinars or pulvinari nucleus or nucleus oral pulvinar or nucleus pulvinar or pulvinar thalami or nucleus pulvinari or nucleus lateral pulvinar or thalami pulvinar or nucleus inferior pulvinar or pulvinaris nucleus or nucleus pulvinaris or pulvinar thalamus or inferior pulvinar nucleus or pulvinar nucleus lateral or Mediodorsal Thalamic Nucleus or medialis dorsali nucleus or medial dorsal thalamic nucleus or mediodorsal nucleus or dorsomedialis thalamus nucleus or nucleus dorsomedial thalamic or nuclei medial thalamic or dorsali nucleus medialis or nucleus mediodorsal or nucleus dorsomedialis thalamus or nucleus medialis dorsali or nucleus mediodorsal thalamic or thalami nucleus dorsomedialis or thalamus nucleus dorsomedialis or thalamic nucleus medial or dorsomedialis thalami nucleus or thalamic nuclei medial or nucleus medial thalamic or mediodorsal thalamic nucleus or nucleus dorsomedial or nucleus medialis dorsalis or thalamic nucleus mediodorsal or dorsal medial nucleus or medialis dorsalis nucleus or nucleus dorsomedialis thalami or medial thalamic nucleus or Midline Thalamic Nuclei or parataenial nucleus or nucleus reunien or rhomboid nucleusor nucleus subfascular or nuclear group midline or paratenial nucleus or rhomboidal nucleus or rhomboid thalamic nucleus or nucleus rhomboid thalamic or reuniens nucleus or subfascular nucleus or thalamus nucleus reuniens or thalami nucleus reuniens or nucleus paraventricular thalamic or reunien nucleus or reuniens thalami nucleusor paraventricular nucleus of thalamus or paraventricular thalamic nucleus or midline thalamic nucleus or thalamic nuclei midline or paratenial thalamic nucleus or thalamic nucleus rhomboid or periventricular nuclei of thalamus or thalamic nucleus reuniens or reuniens thalamus nucleus or thalamus midline nucleus or nucleus paratenial or thalamus paraventricular nucleus or midline thalamic nuclei or thalamic nucleus subfascular or nucleus reuniens thalamus or thalamic nucleus paratenial or nucleus reuniens or nucleus rhomboid or thalamic nucleus paraventricular or midline nuclear group or Posterior Thalamic Nuclei or supergeniculate nucleus or posterior nuclear complicesor nucleus supergeniculateor posterior thalamic nuclei or suprageniculate thalamic nucleus or submedial nucleus or limitans nucleus or thalamic nuclei posterior or nucleus limitan or thalamic nucleus suprageniculate or nucleus submedial or nuclear complices posterior or complices posterior nuclear or posterior nucleus of thalamus or nucleus limitans or nucleus suprageniculate thalamic or posterior thalamic nucleus or posterior thalamic nuclear group or posterior nuclear complex or nuclear complex posterior or thalamus posterior nucleus or Ventral Thalamic Nuclei or posterior nucleus ventral or ventrolateral thalamic nucleus or intermedius nucleus ventralis or ventral posterior nucleus or ventralis intermedius nucleus or ventrobasal complex or ventralis posteromediali nucleus or nuclear mass ventral or group ventral nuclear or ventralis posterolateralis nucleus or nucleus ventral anterior or ventral nuclear groups or laterali nucleus ventralis or nucleus ventral posterolateral or ventralis posterior nucleus or masses ventral nuclear or nucleus ventralis posterolaterali or ventral lateral nucleus or nucleus ventralis intermedius or ventral anterior thalamic nucleus or thalamic nucleus ventral or posterolaterali nucleus ventralis or ventral posteromedial thalamic nucleus or nucleus ventrolateralis thalamus or ventrobasal complices or nucleus ventralis posteromedialis or nuclei ventral thalamic or nucleus ventrolateralis thalami or mass ventral nuclearor ventrolateralis thalami nucleus or ventrolateralis thalamus nucleus or posterolateral nucleus ventral or nuclear group ventral or arcuate nucleus 3 or nucleus ventralis posteriors or ventral posterior thalamic nucleus or ventral posterior medial nucleus or ventral posteroinferior nucleus or posteroinferior nucleus ventral or posteriors nucleus ventralis or arcuate nucleus-3 or thalamus nucleus ventrolateralis or nucleus ventralis or posteromediali or complex ventrobasal or ventral lateral thalamic nucleus or ventral thalamic nuclei or ventral lateral thalamic nuclei or ventral posteromedial nucleus or posteromedialis nucleus ventralis or ventral anterior nucleus or ventral posterolateral nucleus or nuclear masses ventral or ventral posterior inferior thalamic nucleus or thalamic nucleus ventrolateral or ventral nuclear group or thalamus ventrolateral or thalami nucleus ventrolateralis or posteromediali nucleus ventralis or posterolateralis nucleus ventralis or posterior nucleus ventralis or nucleus ventral posteromedial or nucleus ventralis laterali or ventral posterolateral thalamic nucleus or nucleus ventral thalamic or ventralis lateralis nucleus or ventral nuclear mass or ventralis posteriors nucleus or ventralis laterali nucleus or nucleus ventral posterior or ventral thalamic nucleus or ventrolateral thalamus or nucleus ventralis lateralis or Telencephalon or telencephalon or endbrain or endbrains or Cerebrum or cerebrum or cerebral hemisphere left or cerebral hemisphere right or cerebral hemispheres or right cerebral hemisphere or cerebral hemisphere or left cerebral hemisphere or Basal Ganglia or ganglia basal or nuclei basal or basal ganglia or ganglion basal or basal nuclei or claustrum or Corpus Striatum or lenticular nucleus or nucleus lentiform or lentiformis nucleus or lentiform nucleus or corpus striatum or nucleus lenticular or nucleus lentiformis or lentiform nuclei or striatum corpus or nuclei lentiform or Globus Pallidus or pallidum or paleostriatum or globus pallidus or pallidums or Neostriatum or Caudate Nucleus or nucleus caudatus or caudate nucleus or caudatus nucleusor nucleus caudate or caudatus or High Vocal Center or Putamen or putamens or nucleus putamens or putamens nucleus or putamen nucleus or nucleus putamen or putamen or Ventral Striatum or Nucleus Accumbens or nucleus accumbens or accumbens septus nucleus or accumbens septi nucleusor nucleus accumbens septi or septi nucleus accumbens or accumbens nucleus or septus nucleus accumbens or nucleus accumbens septus or Olfactory Tubercleor Islands of Calleja or Basal Nucleus of Meynert or nucleus basalis of meynert or meynert basal nucleus or nucleus basalis magnocellularis or basal nucleus of meynert or meynert nucleus basalisor Cerebral Cortex or plates cortical or insular cortex or cerebral cortices or archipalliums or paleocortex or allocortices or periallocortices or plate cortical or cerebri cortex or cortices cerebral or paleocortices or cortices insular or insular cortices or cortex insular or periallocortex or archipallium or cortical plates or cortex cerebral or cortex cerebri or reil insula or cortex cerebrus or cortical plate or Frontal Lobe or gyrus anterior centralor central gyrus anterior or lobe frontalor frontal lobeor cortex frontal or gyrus precentralis or frontal eye fieldor supplementary eye field or gyrus precentrali or frontali lobusor precentrali gyrus or frontal lobes or frontal cortex or field supplementary eye or lobes frontal or eye field  supplementary or lobus frontali or supplementary eye fields or frontalis lobus or gyrus precentral or eye fields supplementary or eye fields frontal or anterior central gyrus or fields frontal eye or lobus frontalis or Motor Cortex or motor area or primary motor cortex or motor area precentral or strip motor or somatomotor areas or strips motor or motor cortices primary or premotor areas or motor area secondary or cortex precentral motor or motor area somatic or supplementary motor areas or area primary motoror area premotor or secondary motor area or motor cortices secondary or area motor or secondary motor areas or area somatomotor or motor areas or motor cortex secondary or precentral motor areas or cortices secondary motor or area supplementary motor or motor areas supplementary or area precentral motor or cortices primary motor or precentral motor cortices or areas somatic motor or area somatic motor or areas motor or motor cortex precentral or motor areas precentral or motor strips or cortex primary motor or somatomotor area or premotor area or precentral motor cortex or primary motor area or somatic motor area or motor areas somatic or areas premotor or areas somatomotor or areas precentral motor or areas supplementary motor or motor cortex primary or cortex secondary or motor primary motor cortices or motor cortex or motor cortices precentral or motor area supplementary or cortices precentral motor or somatic motor areas or cortex motor or areas secondary motor or Prefrontal Cortex or orbital gyrus) | | |  | | | | |  |
| #13 | (hippocampal mossy fibers or mossy fiber hippocampal or Fornix, Brain or hippocampal commissure or hippocampal commissures or  commissures dorsal hippocampal or fornix commissures or fornices or brain fimbrias or fornical commissures or fornical commissure or fornix or hippocampal commissures dorsal or commissures hippocampal or fornix-fimbria or hippocampal commissure dorsal or fimbria or fornix fimbria or fimbria of hippocampus or brain fornices or dorsal hippocampal commissure or commissure fornical or commissure dorsal hippocampal or commissure of fornix or commissures fornical or commissure hippocampal or fornix commissure or fimbria-fornix or fimbria fornix or fimbria brain or hippocampus fimbrias or hippocampus fimbria or brain fimbria or Hypothalamus or preoptico-hypothalamic areas or preoptico hypothalamic area or lamina terminalis or hypothalamus or areas preoptico-hypothalamic or area preoptico-hypothalamic or preoptico-hypothalamic area or Hypothalamic Area, Lateral or area hypothalamica laterali or hypothalamica  laterali area or hypothalami area lateralis or lateralis area hypothalamica or hypothalamus area lateralis or laterali area hypothalamica or areas lateral hypothalamic or lateralis hypothalami area or lateral hypothalamic areas or accessory nucleus of the ventral horn or lateral tuberal nuclei or tuberal nucleus lateral or lateral hypothalamus or area hypothalamica lateralis or hypothalamus lateral or tuberomammillary nucleus or hypothalamic area lateral or nucleus tuberomammillary or nuclei lateral tuberal or nucleus lateral hypothalamic or lateralis hypothalamus area or area lateral hypothalamic or hypothalamic nucleus lateral or area lateralis hypothalamus or nucleus lateral tuberal or Hypothalamus, Anterior or commissures anterior hypothalamic or anterior hypothalamic decussation of ganser or hypothalamic commissures anterior or anterior hypothalamic commissures or commissure anterior hypothalamic or periventricular nucleus anteroventral or nucleus anteroventral periventricular or anterior hypothalamic commissure or hypothalamic commissure anterior or hypothalamus anterior or hypothalamus supraoptic or anteroventral periventricular nucleus or anterior hypothalamus or supraoptic hypothalamus or Anterior Hypothalamic Nucleus or areas anterior hypothalamic or hypothalamic area anterior or nucleus anterior hypothalamic or anterior hypothalamic nucleus or hypothalami nucleus anterior or hypothalamic areas anterior or anterior hypothalami nucleus or anterior hypothalamic area or area anterior hypothalamic or nucleus anterior hypothalamus or hypothalamus nucleus anterior or anterior hypothalamic areas or anterior hypothalamus nucleus or nucleus anterior hypothalami or hypothalamic nucleus anterior or Organum Vasculosum or Paraventricular Hypothalamic Nucleus or hypothalamic paraventricular nucleus or paraventricular hypothalamic nucleus or nucleus paraventricular hypothalamic or nucleus hypothalamic paraventricular or nucleus paraventricular or paraventricular nucleus or hypothalamic nucleus paraventricular or paraventricular nucleus hypothalamic or Preoptic Area or area medial preoptic or preoptic area medial or preoptic nucleus or nuclei preoptic or lateral preoptic area or preoptic areas lateral or area preoptic or areas medial preoptic or area lateral preoptic or preoptic areas medial or lateral preoptic areas or preoptica area or nucleus preoptic or medial preoptic areas or areas lateral preoptic or area preoptica or areas preoptic or preoptic nuclei or medial preoptic area or preoptic area or preoptic areas or Suprachiasmatic Nucleus or nucleus suprachiasmatic or suprachiasmatic nucleus or Supraoptic Nucleus or hypothalamus supraoptic nucleus or supraoptic group accessory or accessory supraoptic groups or supraoptic nucleus of hypothalamus or supraopticus nucleus or groups accessory supraoptic or nucleus supraoptic or group accessory supraoptic or accessory supraoptic group or nucleus supraopticus or supraoptic groups accessory or supraoptic nucleus or Hypothalamus, Middle or regions intermediate hypothalamic or hypothalamic region intermediate or region intermediate hypothalamic or middle hypothalamus or hypothalamus medial or hypothalamic regions intermediate or intermediate hypothalamic regions or intermediate hypothalamic region or hypothalamus middle or medial hypothalamus or Arcuate Nucleus of Hypothalamus or nucleus arcuate or arcuate nucleus or hypothalamus arcuate nucleus or nucleus infundibular or infundibular nucleus or arcuate nucleus of hypothalamus or Dorsomedial Hypothalamic Nucleus or nucleus arcuate or arcuate nucleus or hypothalamus arcuate nucleus or nucleus infundibular or infundibular nucleus or arcuate nucleus of hypothalamus or Hypothalamo-Hypophyseal System or hypothalamic pituitary unit or hypothalamo hypophyseal system or hypothalamo-hypophyseal system or hypothalamic-pituitary unit or Median Eminence or eminentia medianas or median eminence or eminences medial or eminence medial or medial eminences or medianas eminentia or eminentia mediana or mediana eminentia or eminence median or medial eminence or Pituitary Gland or hypophyseal infundibulum or infundibular hypothalamus or pituitary glands or infundibulum or stalk infundibular or  hypothalamus infundibular or infundibulums or pituitary stalks or pituitary gland or hypophysis or pituitary stalk or infundibular stem or stalks infundibular or glands pituitary or hypophysis cerebri or hypophyseal stalks or cerebri hypophysis orstalk hypophyseal or infundibular stalk or infundibular stalks or hypophysis cerebrus or hypophyseal stalk or Pituitary Gland, Anterior or lobus anteriors or anterior lobe of pituitary or anterior lobus or pituitary pars distalis or anterior pituitary glands or anteriors lobus or lobus anterior or pituitary gland anterior or adenohypophyses or pituitary glands anterior or adenohypophysis or pituitary anterior lobe or anterior pituitary gland or pars distalis of pituitary or Corticotrophs or Gonadotrophs or lh producing cells or lh-secreting cells or fsh cells or gonadotrophs or lh cell or fsh-secreting cellsor fsh secreting cells or fshproducing cells or fsh-producing cell or fsh cell or lh-producing cells or fsh producing cells or lh secreting cells or fsh-secreting cell or gonadotroph or lh-producing cell or lh-secreting cell or lh cell or Lactotrophs or pituitary prolactin-secreting cells or lactotrophs or pituitary prolactin cell or prolactin-secreting cell pituitary or prolactin-secreting cells pituitary or lactotroph or prolactin cell pituitary or prolactin cells pituitary or pituitary prolactin cells or pituitary prolactin-secreting cell or pituitary prolactin secreting cells or Somatotrophs or gh cell pituitary or somatotrophs or gh cells pituitary or pituitary growth hormone-secreting cells or pituitary gh cell or pituitary growth hormone secreting cells or pituitary gh cells or somatotroph or Thyrotrophs or Pituitary Gland, Intermediate or Melanotrophs or Pituitary Gland, Posterior or lobes neural or posterior pituitary glands or neural lobe or pituitary pars nervosa or infundibular processes or infundibular process or process infundibular or neurohypophysis or lobe neural or gland posterior pituitary or pituitary posterior lobe or pars nervosa of pituitary or posterior lobe of pituitary or neural lobes or nervosus lobus or lobus nervosus or pituitary gland posterior or processes infundibular or Tuber Cinereum or cinereums tuber or cinereum tuber or tuber cinereum or tuber cinereums or Ventromedial Hypothalamic Nucleus or nucleus ventromedial hypothalamic or hypothalamic nucleus ventromedial or ventromedial hypothalamic nucleus or Hypothalamus, Posterior or posteriors area hypothalamica or area hypothalamica posterior or mammillary regions or region mammillary or nucleus posterior periventricular or hypothalamic regions posterior or hypothalamus posteriors or mammillary region or posterior area hypothalamica or posterior hypothalamic regions or supramammillary commissures or region posterior hypothalamic or supramammillary commissure or regions posterior hypothalamic or posterior hypothalamus or commissures supramammillary or premammillary nucleus or hypothalamic region posterior or posterior hypothalamic region or commissure supramammillary or hypothalamus posterior or hypothalamica posteriors area or periventricular nucleus posterior or nucleus premammillary or Mammillary Bodies or mammillary bodies ormammillary body or body mammillary or mamillary bodies or body mamillary or bodies mamillary or bodies mammillary or mamillary body or Limbic Lobe or Gyrus Cinguli or gyrus cingular or anterior cingulate gyrus or cingulate gyri posterior or cortex anterior cingulate or posterior cingulate gyrus or cinguli anteriors gyrus or mesial region superior or gyrus cingulate or cingulate cortex anterior or cingulate cortex or superior mesial regions or regions cingulate or cortex posterior cingulate or anterior cingulate cortices or posterior cingulates or cingulate bodies or cingulates anterior or cortices anterior cingulate or posterior cingulate cortices or mesial regions superior or posterior cingulate cortex or regions posterior cingulate or cingulate posterior or posterior cingulate region or region posterior cingulate or body cingulate or cortex cingulate or posterior cingulate regions or  cingulate gyrus anterior or cingulate gyrus or cingulate gyrus posterior or cingular gyrus or bodies cingulate or cingulate area or anterior cingulates or area cingulate or cingulate regions or regions superior mesial or ingulates posterior or areas cingulate or cingulate cortices anterior or anterior gyrus cinguli or gyri posterior cingulate or gyrus anterior cingulate or gyrus cinguli anteriors or cinguli anterior gyrus or superior mesial region or anterior cingulate or gyrus cinguli anterior or cingulate anterior or region cingulate or cingulate areas or Parahippocampal Gyrus or gyrus parahippocampal or gyri parahippocampal or parahippocampal gyri posterior or hippocampal gyrus or gyri posterior parahippocampal or posterior parahippocampal gyrus or gyrus parahippocampalis or parahippocampal gyrus uncus or presubiculums or posterior parahippocampal gyri or gyrus posterior parahippocampal or parahippocampal gyrus posterior or uncus of parahippocampal gyrus or gyri hippocampal or parahippocampal gyrus or presubiculum or gyrus hippocampi or uncus parahippocampal gyrus or gyrus uncus parahippocampal or gyrus hippocampal or parahippocampal gyri or Entorhinal Cortex or area entorhinali or areas entorhinal or entorhinalis area or entorhinal area or area entorhinal or entorhinal cortices or area entorhinalis or cortices entorhinal or entorhinali area or olfactory cortices secondary or secondary olfactory cortex or cortex secondary olfactory or cortices secondary olfactory or entorhinal cortex or olfactory cortex secondary or secondary olfactory cortices or cortex entorhinal or entorhinal areas or Olfactory Pathways or olfactory pathways or pathways olfactory or olfactory pathway or pathway olfactory or Perforant Pathway or pathway perforant or pathways perforant or perforant paths or perforant pathways or perforant pathway or fasciculus perforating or paths perforant or perforant path or perforating fasciculus or path perforant or Septum of Brain or paraterminal body or brain septums or brain septum or septum of brain or paraterminal bodies or area septal or bodies paraterminal or body paraterminal or septal area or region septal or septal region or Septal Nuclei or nucleus of the stria terminalis or septi lateralis nucleus or septal nuclear complices or nucleus of anterior commissure or terminali nucleus striae or laterali nucleus septalis or nucleus lateralis septi or nucleus lateralis septus or nuclear complices septal or septofimbrial nucleus or diagonal band nucleus or nucleus septofimbrial or nucleus septi lateralis or laterali nucleus septi or nucleus triangular septal or medial septal nucleus or nucleus of diagonal band or nucleus septalis lateralis or nucleus striae terminali or nuclear complex septal or septum nucleus lateral or lateral septal nucleus or lateralis nucleus septalis or septal nucleus lateral or septalis laterali nucleus or nuclei septal or anterior commissure nucleus or septus nucleus lateralis or septi laterali nucleus or nucleus medial septum or nucleus septalis laterali or triangularis septus nucleus or lateralis nucleus septi or complex septal nuclear or nucleus striae terminalis or nucleus of stria terminalis or septalis lateralis nucleus or dorsal septal nucleus or nucleus triangularis septus or nucleus lateral septumor nucleus triangularis septi or nucleus lateral septal or septal nucleus triangular or terminalis nucleus striae or septi nucleus lateralis or septi nucleus triangularis or triangular septal nucleus or septus nucleus triangularis or nucleus medial septal or complices septal nuclear or lateralis septus nucleus or medial septum nucleus or lateralis septi nucleus or Substantia Innominata or innominata substantia or substantia innominata or Prosencephalon or prosencephalon or forebrains or forebrain or Diencephalon or diencephalon or interbrain or interbrains or Optic Chiasm or chiasmas optic or optic chiasms or decussation optic or chiasma optic or optic chiasm or optic chiasma or optic decussation or opticums chiasma or optic decussations or chiasma opticum or decussations optic or opticum chiasma or optic chiasmas or chiasma opticums or chiasms optic or chiasm optic or Optic Tract or Subthalamus or subthalamus or fasciculus thalamic or field h nucleus or campi forelus nucleus or fasciculus lenticular or field h1 forel's or campi foreli nucleus or enticular fasciculus or forels field h2 or forel field h2 or thalamicus fasciculus or fasciculus thalamicus or thalamic fasciculus or forelus nucleus campi or nucleus of ansa lenticularis or foreli nucleus campi or nucleus campi forelus or nucleus of field h or forels field h1 or forel's field h2 or field h1 of forel or forel field h1 or Entopeduncular Nucleus or Subthalamic Nucleus or nucleus of luys or luys subthalamic nucleus or corpus luysi or luys body or subthalamic nucleus of luys or subthalamicus nucleus or luys nucleus or nucleus subthalamic or luysi corpus or body of luys or nucleus subthalamicus or subthalamic nucleus or Zona Incerta or Thalamus or thalamencephalon or thalamencephalons or thalamus or Thalamic Nuclei or nuclei thalamic or thalamic nuclei or Anterior Thalamic Nuclei or nucleus anterodorsal thalamic or anterior nuclear group or nucleus anteromedial thalamic or nucleus anteroventral thalamic or thalamus anterior nucleus or anterior thalamic nucleus or nucleus anteroventral or anteroventral nucleus or thalamic nucleus anterodorsal or nuclei anterior thalamic or thalamic nuclei anterior or anteromedial nucleus or anteromedial thalamic nucleusor thalamus anterior or nucleus anteromedial or anterodorsal nucleus or anterior thalamus or anterior thalamic nuclei or anterodorsal thalamic nucleus or nucleus anterodorsal or thalamic nucleus anteroventral or Geniculate Bodies or nucleus geniculate or medial geniculate nucleus or geniculate complex medial or geniculatum mediales corpus or bodies geniculate or nucleus lateral geniculate or mediales corpus geniculatum or geniculate bodies medial or mediale corpus geniculatum or geniculate body or geniculatum mediale corpus or geniculate nucleus lateral or geniculate bodies or geniculate bodies lateral or metathalamus or corpus geniculatum mediale or geniculate body lateral or complex medial geniculate or nucleus geniculatus lateralis pars dorsalis or geniculate body medial or geniculate complices medial or geniculate nucleus or complices medial geniculate or medial geniculate body or medial geniculate bodies or geniculate nucleus medial or Intralaminar Thalamic Nuclei or nucleus paracentrali or centrum medianum or paracentrali nucleus or centromedian thalamic nucleus or central lateral nucleus or thalamic nucleus parafascicular or central lateral thalamic nucleus or parafascicular thalamic nucleus or thalamic nucleus intralaminar or nucleus central dorsal or parafascicular nucleus of the thalamus or centromedian nucleus or intralaminar nuclei rostral or intralaminar nuclear group or thalamic nucleus centromedian or parafascicularis nucleus or nucleus central lateral or thalamic nuclei intralaminar or central dorsal thalamic nucleus or interlaminar nuclei of thalamus or rostral intralaminar nuclei or thalamus nucleus parafascicularis or centrum medianums nucleus or medianum centrum or thalamic nucleus paracentral or thalamus reticulate nucleus or nucleus paracentral or nucleus central medial or paracentral thalamic nucleus or median nucleus centre or nuclei intralaminar thalamic or nuclei rostral intralaminar or  central medial nucleus or nucleus centrum medianums or nucleus centre median or medianum nucleus centrum or nucleus paracentral thalamic or nucleus centromedian thalamic or nucleus parafascicularis thalamus or nucleus intralaminar thalamic or nucleus centrum medianum or nucleus parafascicularis thalami or parafascicularis thalami nucleus or parafascicularis thalamus nucleus or reticulate nuclei of thalamus or nucleus parafasciculari or centrum medianums or centrum medianum nucleus or paracentralis nucleus or lateral nucleus central or parafascicular nucleus or central medial thalamic nucleus or nucleus centromedian or Lateral Thalamic Nuclei or medial pulvinar nucleus or Pulvinar or anterior pulvinar nucleus) | | |  | | | | |  |
| #12 | (Brain* or hippocamp* or encephalon or Blood Brain Barrier or hemato-encephalic barriers or barriers brain-blood or hemato encephalic  barrier or barriers hemato-encephalic or barrier hemato-encephalic or hemato-encephalic barrier or truncus cerebrus or truncus cerebri or cerebri truncus or brainstems or cerebrus truncus or Mesencephalon or mesencephalon or mesencephalons or midbrains or midbrain or Cerebral Peduncle or Cerebral Crus or Substantia Nigra or nigras substantia or nigra substantia or substantia nigras or Pars Compacta or Pars Reticulata or Tegmentum Mesencephali or midbrain trigeminal nucleus or nucleus peripeduncular or annulari nucleus or nervi trochlearis nucleus or midbrain tegmentum or mesencephalus tegmentum or tegmental nucleus ventral or mesencephalic tegmentums or midbrain tegmentums or trigeminal nucleus mesencephalic or tegmentums midbrain or trochlearis nucleus nervi or nucleus annularis or trigeminal nucleus midbrain or nucleus annular or mesencephali tegmentum or darkshevichs nucleus or tegmentums mesencephalic or ventral tegmental nucleus or mesencephalic trigeminal nucleus or nervi trochleari nucleus or nucleus darkshevich's or darkschewitsch nucleus or tegmentum of midbrain or nucleus annulari or cajal interstitial nucleus or mesencephalic tegmentum or nuclei accessory oculomotor or trochlear nucleus or annularis nucleus or nucleus mesencephalic trigeminal or nucleus of darkschewitschor peripeduncular nucleus or oculomotor nuclei accessory or tegmentum midbrain or tegmentum mesencephali or nucleus nervi trochlearis or darkshevich nucleus or nucleus tractus mesencephalici nervi trigemini or interstitial nucleus of cajal or Cerebral Aqueduct or ducts mesencephalic or mesencephalic ducts or aqueduct mesencephalic or sylvian aqueducts or duct mesencephalic or sylvius aqueduct or cerebrus aqueductus or aqueductus cerebrus or cerebral aqueduct or aqueduct sylvian or aqueduct of sylvius or mesencephalic duct or cerebral aqueducts or aqueducts sylvian or aqueduct cerebral or sylvian aqueduct or aqueductus cerebri or aqueducts mesencephalic or cerebri aqueductus or mesencephalic aqueduct or Midbrain Reticular Formation or Pedunculopontine Tegmental Nucleus or nucleus tegmentalis pedunculopontinus or nucleus pedunculopontine tegmental or tegmental nucleus pedunculopontine or pedunculopontine tegmental nucleus or Oculomotor Nuclear Complex or Edinger-Westphal Nucleus or Periaqueductal Grey or greys central periaqueductal or griseum centrales or central grey substance of midbrain or periaqueductal greys central or grey matter periaqueductal or grey central periaqueductal or substantia grisea centralis or periaqueductal grey matter or central periaqueductal grey or grisea centralis substantia or periaqueductal grey or centrale mesencephali griseumor centrale mesencephalus griseum or centrale griseum or grey matters periaqueductalor centrales griseum or periaqueductal grey central or substantia grisea centralis mesencephali or mesencephalus griseum central or midbrain central grey or central grey mesencephalic or central periaqueductal greys or central grey midbrain or griseum centrale mesencephali or Raphe Nuclei or nucleus incertus or nucleus superior central ornuclei raphe ornucleus interfascicular or superior central nucleus or raphe nuclei or interfascicular nucleus or raphe nucleus or incertus nucleus or central nucleus superior or rostral linear nucleus of the raphe or caudal linear nucleus of the raphe or rostral linear nucleus of raphe or nucleus rapheor Dorsal Raphe Nucleus or Interpeduncular Nucleus or Midbrain Raphe Nuclei or Red Nucleus or nucleus ruber or red nucleus or nucleus red or Ventral Tegmental Area or tegmentalis ventralis area or tegmentalis ventrali area or area tegmentalis ventralis or ventral tegmental area of tsai or ventral tegmental area or tegmental area ventral or area tegmentalis ventrali or Locus Coeruleus or coeruleus complex locus or complices locus coeruleus or locus caeruleus or complex locus ceruleus or complices locus ceruleus or coeruleus complices locus or ceruleus complex locus or locus ceruleus complex or complex locus coeruleus or locus ceruleus complices or locus ceruleus or nucleus pigmentosus pontis or locus coeruleus complices or pontis nucleus pigmentosus or ceruleus complices locus or locus coeruleus or locus coeruleus complex or Tectum Mesencephali or corpora quadrigemina or inferior colliculus commissures or colliculus commissures superior or colliculus commissures inferior or quadrigeminal plates or superior colliculus commissure or plate quadrigeminal or commissure of superior colliculus or quadrigemina corpora or commissure of inferior colliculus or lamina quadrigemina or inferior colliculus commissure or colliculus commissure inferior or quadrigeminal plate or tectum mesencephalus or mesencephalus tectum or plates quadrigeminal or quadrigemina lamina or colliculus commissure superior or Inferior Colliculi or colliculi inferior or inferior colliculi or inferiors colliculus or posterior colliculus or brachial nucleus of the inferior colliculus or caudal colliculus or colliculus inferiors or colliculus caudal or inferior colliculus or colliculus posterior or colliculus inferior or Subcommissural Organ or subcommissural organs or subcommissural organ or organs subcommissural or organ subcommissural or Superior Colliculi or mammalian optic lobesor optic lobe mammalian or optic tectums or superior colliculi or optic tectum or anterior colliculus or colliculus superior or human optic lobes or superior colliculus or optic lobes human or optic lobes mamalian or optic lobe human or colliculi superior or tectum optic or tectums optic or mammalian optic lobe or human optic lobe or colliculus anterior or Reticular Formation or formations reticular or reticular formation or reticular formations or formation reticular or edunculopontine Tegmental Nucleus or nucleus tegmentalis pedunculopontinus or nucleus pedunculopontine tegmental or tegmental nucleus pedunculopontine or pedunculopontine tegmental nucleus or Respiratory Center or centers respiratory or respiratory centers or center respiratory or respiratory center or hombencephalon or hind brains or brains hind or rhombencephalons or hindbrain or hindbrains or brain hind or rhombencephalon or hind brain or Medulla Oblongata or medulla oblongata or nucleus ambiguous or arcuate nucleus-1 or accessory cuneate nucleus or nucleus external cuneate or cuneate nucleus accessory or nucleus ambiguous or medulla oblongatas or arcuate nucleus of the medulla or cuneate nucleus lateralor nucleus lateral cuneate or ambiguous nucleus or cuneate nucleus external or arcuate nucleus 1 or external cuneate nucleus or ambiguus nucleus or arcuate nucleus-1s or lateral cuneate nucleus or Area Postrema or area postremas or trigger zone chemoreceptor or chemoreceptor trigger zone or chemoreceptor trigger zones or trigger zones chemoreceptor or zone chemoreceptor trigger or postrema area or zones chemoreceptor trigger or area postrema or Olivary Nucleus ornucleus basalis olivary or nucleus olivary or basalis olivary nucleus or nucleus olivary basal or olivary basal nucleus or basal nucleus olivary or olivary nucleus or Raphe nuclei or nucleus incertus or nucleus superior central or nuclei raphe or nucleus interfascicular or superior central nucleus or raphe nuclei or interfascicular nucleus or raphe nucleus or incertus nucleus or central nucleus superior or rostral linear nucleus of the raphe or caudal linear nucleus ofthe raphe or rostral linear nucleus of raphe or nucleus raphe or Nucleus Raphe Obscurus or Nucleus Raphe Pallidus or Solitary Nucleusor solitary nuclear complices or nucleus of tractus solitaries or complex solitary nuclear or tractus solitarii nuclei or nucleus solitaries or solitarius nucleus tractus or tractus solitarius nucleus or solitariusnuclei tractus or solitary tract nucleus or nucleus solitary tract or solitary nuclear complex or tractus solitarius nuclei or nuclear  complices solitary or nuclei tractus solitarii or solitary nucleus ornucleus solitaryor nucleus of the solitary tract or nuclear complex solitary or complices solitary nuclear or nucleus of solitary tract or nucleus tractus solitaries or Trigeminal Nucleus, Spinal or trigeminal nucleus spinal or nucleus spinal trigeminal or spinal trigeminal nucleus or Trigeminal Caudal Nucleus or caudal nucleus trigeminal or nucleus trigeminal caudal or trigeminal caudal nucleus or Metencephalon or Cerebellumor corpus cerebellus or parencephalons or cerebellus corpus or cerebellum or cerebellums or corpus cerebelli or parencephalon or cerebelli corpus or Cerebellar Cortex or cerebelli cortex or cortex cerebellus or cerebellar cortex or cortex cerebelli orcerebellus cortex orcortex cerebellar or Cerebellar Vermis or Purkinje Cells or purkinje cells or cells purkinje or Cerebellar Nuclei or nucleus dentatus or Cerebellopontine Angle or central nucleus or central nucleus or interposed nucleus anterior or nucleus globosus or medial cerebellar nucleus or emboliformis nucleus or nuclei cerebellar or intracerebellar nuclei or nucleus fastigii or nucleus fastigial orfastigii nucleus or central nuclei or nuclei central or deep cerebellar nucleus or intracerebellar nucleus or nucleus fastigial cerebellar or nucleus anterior interposed or nucleus intracerebellar oranterior interposed nucleus or nucleus anterior interpositus or nucleus medial cerebellar or nuclei intracerebellar or nucleus dentate or dentate nucleus or interpositus nucleus anterior or globosus nucleus orcerebellar nucleus deep or nucleus central or nucleus cerebellar or cerebellar nuclei deep or nucleus dentate cerebellar or anterior interpositus nucleus or cerebellar nucleus medial or cerebellar nuclei or fastigial cerebellar nucleus or Pons or pons or varolii ponsor pontes or pons varolius or varolius pons or pons varolii or ponte or Barrington's Nucleusor Cochlear Nucleus or cochlear nucleus or nuclei cochlear or cochlear nuclei or nucleus cochlear or Kolliker-Fuse Nucleus or Middle Cerebellar Peduncle or Pontine Tegmentum or Abducens Nucleus or Facial Nucleus or Parabrachial Nucleus or Nucleus Raphe Magnus or Superior Olivary Complex or Trapezoid Body or Trigeminal Motor Nucleus or Vestibular Nuclei or schwalbes nucleus or nucleus schwalbe or vestibular nuclei or vestibular nucleus medial or nuclei vestibular or schwalbe's nucleus or nucleus schwalbe's or medial vestibular nucleus or schwalbe nucleus or nucleus medial vestibular or Vestibular Nucleus, Lateral or deiters nucleus or deiter's nucleus or nucleus of deiters or lateral vestibular nucleus or nucleus lateral vestibular or vestibularis laterali nucleus or nucleus vestibularis laterali or vestibular nucleus lateral or vestibularis magnocellulari nucleus or vestibularis magnocellularis nucleus or deiter nucleus or nucleus vestibularis magnocellularis or nucleus vestibularis magnocellulari or nucleus deiter or nucleus vestibularis lateralis or vestibularis lateralis nucleus or nucleus deiter's or Tectospinal Fibers or Trigeminal Nuclei or trigeminal nucleus or trigeminal nuclear complices or trigeminal nuclear complex or nuclei trigeminal or trigeminal nuclei or nucleus trigeminal or nuclear complices trigeminal or nuclear complex trigeminal or Grey Matter or grey matter or grey matters cerebellar or grey matter cerebellar or matters grey or matter cerebellar grey or grey matter cerebellar or cerebellar grey matters or grey matters or matter cerebellar grey or cerebellar grey matter or matters grey or cerebellar grey matters or matters cerebellar grey or grey matters cerebellar or matters cerebellar grey or grey matter or cerebellar grey matter or matter grey or matter grey or White Matter or white matter cerebellar or matter cerebellar white or matter white or matters cerebellar white or white matters cerebellar or cerebellar white matters or cerebellar white matter or matters white or white matter orwhite matters or Cerebral Ventricles or cerebral ventricle or cerebral ventriclesor monro foramen or ventricles cerebral or foramen of monro or cerebral ventricular system or ventricle cerebral or Choroid Plexus or choroideus plexus or plexus choroideusor choroid plexus or chorioid plexus or plexus chorioid or plexus choroid or Ependyma or ependymal or ependymas or Fourth Ventricle or ventricolo quarto or ventricles fourth or ventricle fourth or 4th ventricle or quarto ventricolos or ventricle 4th or ventricles 4th or fourth ventricle or ventricolos quarto or fourth ventricles or 4th ventricles or quarto ventricolo or Lateral Ventricles or lateral ventricle orsubventricular zones or lateral ventricles or ventricle lateral or zone subventricular or ventricles lateral or subventricular zone or zones subventricular or Septum Pellucidum or septum supracommissural or pelusidum septum or septum pellucidum or lucidums septum or supracommissural septum or pellucidum septum or septum pelusidums or septum pelusidum or pelusidums septum or septum lucidums or supracommissural septums or septums supracommissural or lucidum septum or septum lucidum or Third Ventricle or ventricle or ventricles third or ventricles 3rd or third ventricle or ventricle 3rd or 3rd ventricles or third ventricles or ventricle third or Limbic System or limbic system or system limbic or systems limbic or limbic systems or Amygdala or amygdaloid bodies or corpus amygdaloideums or nucleus intercalated amygdaloid or corpus amygdaloideum or amygdaloid body or complex amygdaloid nuclear or amygdaloid nuclear complices or amygdaloid nucleusor intercalata massa or amygdaloideums corpus or intercalatas massa or amygdaloid nucleus intercalated or nuclear complices amygdaloid or archistriatums or amygdala or massa intercalates or nucleus amygdaloid or amygdaloideum corpus or amygdalae nucleus or nuclear complex amygdaloid or archistriatum or nucleus amygdalae or amygdaloid nuclear complex or Basolateral Nuclear Complex or Central Amygdaloid Nucleus or Corticomedial Nuclear Complex or Periamygdaloid Cortex or epithalamus or Habenula or commissure habenular or habenula complex or habenulas or complices habenula or nucleus habenularis or habenular commissures or complex habenula or habenula complices or nucleus habenular or nucleus habenulari or commissures habenular or habenula or habenularums commissura or commissura habenularum or habenularis nucleus or habenular nuclei or commissura habenularums or nuclei habenular or habenulari nucleus or habenular nucleus or Pineal Gland or pineales corpus or body pineal or glands pineal or pineal glands or pineal body or cerebri epiphysis or corpus pineales or gland pineal or pineale corpus or bodies pineal or corpus pineale or pineal gland or pineal bodies or epiphysis cerebri or Hippocampus or hippocampal formation or propers hippocampus or hippocampus propers or formations hippocampal or horn ammon's or schaffer collateral or ammon horn or hippocampus or horn ammon or cornu ammonis or hippocampus proper or proper hippocampus or collaterals schaffer or formation hippocampal or hippocampal formations or subiculum or subiculums or ammon's horn or CA1 Region, Hippocampal or regio superior of hippocampus or field hippocampus ca1 or ca1 stratum radiatum or stratum radiatum ca1 or hippocampal sector ca1 or hippocampus ca1 field or hippocampus regio superior or ca1 stratum radiatums or sector ca1 hippocampal or ca1 hippocampus or radiatums ca1 stratum or stratum radiatums ca1 or ca1 hippocampal sector or ca1 pyramidal cell area or ca1 region hippocampal or ca1 pyramidal cell layer or ca1 stratum pyramidale or stratum pyramidale ca1 or cornu ammonis 1 area or radiatum ca1 stratum or CA2 Region, Hippocampal or ca2 stratum pyramidale or radiatums ca2 stratum or cornu ammonis 2 area or ca2 field hippocampus or stratum pyramidale ca2 or stratum radiatum ca2 or ca2 stratum radiatums or radiatum ca2 stratum or sector ca2 hippocampal or region hippocampal ca2 or ca2 field of hippocampus or stratum radiatums ca2 or ca2 region hippocampal or hippocampal sector ca2 or hippocampal ca2 region or hippocampus ca2 field or ca2 pyramidal cell layer or field hippocampus ca2 or ca2 pyramidal cell area or CA3 Region, Hippocampal or stratum lucidum ca3 or ca3 stratum lucidum or stratum lucidums ca3 or lucidum ca3 stratum or ca3 region hippocampal or ca3 pyramidal cell area or hippocampus ca3 field or ca3 hippocampal sector or sector ca3 hippocampal or ca3 stratum radiatum or ca3 stratum lucidums or hippocampal ca3 regions or cornu ammonis 3 area or ca3 field of hippocampus or radiatum ca3 stratum or field hippocampus ca3 or stratum radiatums ca3 or ca3 pyramidal cell layer or lucidums ca3 stratum or region hippocampal ca3 or radiatums ca3 stratum or ca3 stratum pyramidale or ca3 field hippocampus or Dentate Gyrus or ca4 region hippocampal or dentate fascia or cornu ammonis 4 area or hilus gyri dentate or ca4 field of hippocampal formation or ca4 hippocampal sector or gyrus dentate or sector ca4 hippocampal or hippocampal ca4 region or area dentata or region hippocampal ca4 or dentata area or field hippocampal ca4 or gyrus dentatus or hilus of the fascia dentata or hilus of dentate gyrus or dentate gyrus or area dentatas or dentata fascia or hippocampal sector ca4 or hippocampal ca4 field or ca4 of lorente de no or Mossy Fibers, Hippocampal or hippocampal mossy fiber or mossy fibers hippocampal) | |  | | | | | |  |
| #11 | (IClH OR "Cl channels" OR "acid-activated Cl channels") | | | | | | | |  |
| #10 | (GJA1 OR GJA3 OR GJA4 OR GJA5 OR GJA6P OR GJA8 OR GJA9 OR GJA10 OR GJB1 OR GJB2 OR GJB3 OR GJB4 OR GJB5 OR GJB6 OR GJB7 OR  GJC1 OR GJC2 OR GJC3 OR GJD2 OR GJD3 OR GJD4 OR GJE1 OR "gap junction protein epsilon 1" OR "gap junction protein delta 4" OR "gap junction protein delta 3" OR "gap junction protein delta 2" OR "gap junction protein gamma 3" OR "gap junction protein gamma 2" OR "gap junction protein gamma 1" OR "gap junction protein beta 7" OR "gap junction protein beta 6" OR "gap junction protein beta 5" OR "gap junction protein beta 4" OR "gap junction protein beta 3"OR "gap junction protein beta 2" OR "gap junction protein beta 1" OR "gap junction protein alpha 10"OR "gap junction protein alpha 9" OR "gap junction protein alpha 8" OR "gap junction protein alpha 5" OR "gap junction protein alpha 4" OR "gap junction protein alpha 3" OR "gap junction protein alpha 1" OR "gap junction protein alpha 6 pseudogene" OR CX43 OR ODD OR SDTY3OR CX46 OR CX37 OR CX40 OR CX50 OR CX58 OR CX59 OR CX62 OR CX32 OR CX26 OR CX31 OR CX30.3 OR CX31.1 OR CX45 OR CX47 OR CX46.6 OR CX30.2 OR CX36 OR CX31.9 OR CX40.1 OR CX23) | | | | |  | | |  |
| #9 | ("cyclic nucleotide gated channel alpha 1" OR "cyclic nucleotide gated channel alpha 2" OR "cyclic nucleotide gated channel alpha 3" OR  "cyclic nucleotide gated channel alpha 4" OR CNGA1 OR CNGA2 OR CNGA3 OR CNGA4 OR "cyclic nucleotide gated channel beta 1" OR "cyclic nucleotide gated channel beta 3" OR CNGB1 OR CNGB3 OR HCN1 OR HCN2 OR HCN3 OR HCN4 OR "hyperpolarization activated cyclic nucleotide gated potassium channel 1" OR "hyperpolarization activated cyclic nucleotide gated potassium channel 3” OR "hyperpolarization activated cyclic nucleotide gated potassium channel 4" OR "hyperpolarization activated cyclic nucleotide gated potassium and sodium channel 2" OR RCNC1 OR RCNCa OR CNG1 OR RP49 OR CNG2 OR OCNC1 OR OCNCa OR OCNCALPHA OR OCNCalpha OR FLJ46312 OR CCNC1 OR CCNCa OR CNG3 OR OCNC2 OR OCNCb OR CNG5 OR RCNC2 OR RCNCb OR GARP OR GAR1 OR CNGB1B OR RP45 OR BCNG-1 OR BCNG-2 OR HAC-2 OR HAC-1 OR KIAA1535) | | | | |  | | |  |
| #8 | ("potassium voltage-gated channel modifier subfamily V member 2" OR "potassium voltage-gated channel modifier subfamily V member 1" OR Kv8.2 OR Kv8.1 OR "potassium voltage-gated channel modifier subfamily S member 3" OR "potassium voltage-gated channel modifier subfamily S member 2" OR "potassium voltage-gated channel modifier subfamily S member 1" OR Kv9.3 OR Kv9.2 OR Kv9.1 OR "potassium voltage-gated channel subfamily Q member 5" OR "potassium voltage-gated channel subfamily Q member 4" OR "potassium voltage-gated channel subfamily Q member 3" OR "potassium voltage-gated channel subfamily Q member 2" OR "potassium voltage-gated channel subfamily Q member 1" OR Kv7.5 OR Kv7.4 OR Kv7.3 OR Kv7.2 OR Kv7.1 OR ENB1 OR BFNC OR KCNA11 OR HNSPC OR KCNA8 OR KVLQT1 OR JLNS1 OR LQT1 OR KCNA9 OR LQT OR EBN OR DFNA2 OR EBN2 OR "potassium voltage-gated channel subfamily H member 8" OR "potassium voltage-gated channel subfamily H member 7" OR "potassium voltage-gated channel subfamily H member 6" OR "potassium voltage-gated channel subfamily H member 5" OR "potassium voltage-gated channel subfamily H member 4" OR "potassium voltage-gated channel subfamily H member 3" OR "potassium voltage-gated channel subfamily H member 2" OR "potassium voltage-gated channel subfamily H member 1" OR LQT2 OR Kv12.1 OR elk3 OR Kv11.3 OR HERG3 OR erg3 OR Kv11.2 OR erg2 OR HERG2 OR Kv10.2 OR H-EAG2 OR eag2 OR Kv12.3 OR Kv12.2 OR Kv11.1 OR elk1 OR elk2 OR BEC1 OR erg1 OR HERG OR Kv10.1 OR eag OR h-eag OR Kv6.4 OR Kv6.3 OR Kv6.2 OR Kv6.1 OR KCNF2 OR kH2 OR K13 OR "potassium voltage-gated channel modifier subfamily G member 4" OR "potassium voltage-gated channel modifier subfamily G member 3" OR "potassium voltage-gated channel modifier subfamily G member 2" OR "potassium voltage-gated channel modifier subfamily G member 1" OR "potassium voltage-gated channel modifier subfamily F member 1" OR KCNF OR Kv5.1 OR "potassium voltage-gated channel subfamily D member 3" OR "potassium voltage-gated channel subfamily D member 2" OR "potassium voltage-gated channel subfamily D member 1" OR Kv4.3 OR Kv4.2 OR Kv4.1 OR "potassium voltage-gated channel subfamily C member 4" OR "potassium voltage-gated channel subfamily C member 3" OR "potassium voltage-gated channel subfamily C member 2" OR "potassium voltage-gated channel subfamily C member 1" OR Kv3.4 OR Kv3.3 OR Kv3.2 OR Kv3.1 OR "potassium voltage-gated channel subfamily B member 2" OR "potassium voltage-gated channel subfamily B member 1" OR Kv2.2 OR Kv2.1 OR "potassium voltage-gated channel subfamily A member 10" OR "potassium voltage-gated channel subfamily A member 7" OR "potassium voltage-gated channel subfamily A member 6" OR "potassium voltage-gated channel subfamily A member 5" OR "potassium voltage-gated channel subfamily A member 4" OR "potassium voltage-gated channel subfamily A member 3" OR "potassium voltage-gated channel subfamily A member 2" OR "potassium voltage-gated channel subfamily A member 1" OR Kv1.8 OR Kv1.7 OR Kv1.6 OR Kv1.5 OR Kv1.4 OR Kv1.3 OR Kv1.2 OR Kv1.1) | | | |  | | | |  |
| #7 | (KCNJ1 OR KCNJ2 OR KCNJ3 OR KCNJ4 OR KCNJ5 OR KCNJ6 OR KCNJ8 OR KCNJ9 OR KCNJ10 OR KCNJ11 OR KCNJ12 OR KCNJ13 OR KCNJ14 OR KCNJ15 OR KCNJ16 OR KCNJ18 OR "potassium voltage-gated channel subfamily J member 1" OR "potassium voltage-gated channel subfamily J member 2" OR "potassium voltage-gated channel subfamily J member 3" OR "potassium voltage-gated channel subfamily J member 4" OR "potassium voltage-gated channel subfamily J member 5" OR "potassium voltage-gated channel subfamily J member 6" OR "potassium voltage-gated channel subfamily J member 8" OR "potassium voltage-gated channel subfamily J member 9" OR "potassium voltage-gated channel subfamily J member 10" OR "potassium voltage-gated channel subfamily J member 11" OR "potassium voltage-gated channel subfamily J member 12" OR "potassium voltage-gated channel subfamily J member 13" OR "potassium voltage-gated channel subfamily J member 14" OR "potassium voltage-gated channel subfamily J member 15" OR "potassium voltage-gated channel subfamily J member 16" OR "potassium voltage-gated channel subfamily J member 18" OR KCNJ7 OR KCNJN1 OR ROMK1 OR Kir1.1 OR Kir2.1 OR IRK1 OR LQT7 OR Kir3.1 OR GIRK1 OR KGA OR Kir2.3 OR HIR OR HRK1 OR hIRK2 OR IRK3 OR Kir3.4 OR CIR OR KATP1 OR GIRK4 OR LQT13 OR Kir3.2 OR GIRK2 OR KATP2 OR BIR1 OR hiGIRK2 OR Kir6.1 OR GIRK3 OR Kir3.3 OR Kir4.1 OR Kir1.2 OR BIR OR Kir6.2 OR Kir2.2 OR Kir2.2v OR IRK2 OR hIRK1 OR Kir7.1 OR Kir1.4 OR LCA16 OR Kir2.4 OR IRK4 OR Kir4.2 OR Kir1.3 OR IRKK OR Kir5.1 OR BIR9 OR KIR2.6 OR TTPP2 OR "Inwardly rectifying potassium channels" OR Kir OR IRK OR "Voltage-gated potassium channels" OR KCNA1 OR KCNA2 OR KCNA3 OR KCNA4 OR KCNA5 OR KCNA6 OR KCNA7 OR KCNA10 OR KCNB1 OR KCNB2 OR KCNC1 OR KCNC2 OR KCNC3 OR KCNC4 OR KCND1 OR KCND2 OR KCND3 OR KCNF1 OR KCNG1 OR KCNG2 OR KCNG3 OR KCNG4 OR KCNH1 OR KCNH2 OR KCNH3 OR KCNH4 OR KCNH5 OR KCNH6 OR KCNH7 OR KCNH8 OR KCNQ1 OR KCNQ2 OR KCNQ3 OR KCNQ4 OR KCNQ5 OR KCNS1 OR KCNS2 OR KCNS3 OR KCNV1OR KCNV2) | | | |  | | | |  |
| #6 | (FLJ14471 OR FEX OR HG38 OR GPR49 OR GPR67 OR GPR48 OR EBI2 OR "G protein-coupled receptor 183" OR "G protein-coupled receptor  182" OR "G protein-coupled receptor 176" OR "G protein-coupled receptor 174" OR "G protein-coupled receptor 173" OR "G protein-coupled receptor 171" OR "G protein-coupled receptor 162" OR "G protein-coupled receptor 161" OR "G protein-coupled receptor 160" OR "G protein-coupled receptor 153" OR "G protein-coupled receptor 152" OR "G protein-coupled receptor 151" OR "G protein-coupled receptor 150" OR "G protein-coupled receptor 149" OR "G protein-coupled receptor 148" OR "G protein-coupled receptor 146" OR "G protein-coupled receptor 142" OR "G protein-coupled receptor 141" OR "G protein-coupled receptor 139" OR "G protein-coupled receptor 135" OR "G protein-coupled receptor 132" OR "G protein-coupled receptor 119" OR "G protein-coupled receptor 101" OR "G protein-coupled receptor 88" OR "G protein-coupled receptor 87" OR "G protein-coupled receptor 85" OR "G protein-coupled receptor 84" OR "G protein-coupled receptor 82" OR "G protein-coupled receptor 79, pseudogene" OR "G protein-coupled receptor 78" OR "G protein-coupled receptor 75" OR "G protein-coupled receptor 68" OR "G protein-coupled receptor 65" OR "G protein-coupled receptor 63" OR "G protein-coupled receptor 62" OR "G protein-coupled receptor 61" OR "G protein-coupled receptor 55" OR "G protein-coupled receptor 52" OR "G protein-coupled receptor 50" OR "G protein-coupled receptor 45" OR "G protein-coupled receptor 42" OR "G protein-coupled receptor 39" OR "G protein-coupled receptor 37" OR "G protein-coupled receptor 35" OR "G protein-coupled receptor 34" OR "G protein-coupled receptor 33" OR "G protein-coupled receptor 32" OR "G protein-coupled receptor 31" OR "G protein-coupled receptor 27" OR "G protein-coupled receptor 26" OR "G protein-coupled receptor 25" OR "G protein-coupled receptor 22” OR "G protein-coupled receptor 21" OR "G protein-coupled receptor 20" OR "G protein-coupled receptor 19" OR "G protein-coupled receptor 18" OR "G protein-coupled receptor 17" OR "G protein-coupled receptor 15” OR "G protein-coupled receptor 12" OR "G protein-coupled receptor 6" OR "G protein-coupled receptor 4" OR "G protein-coupled receptor 3" OR "G protein-coupled receptor 1" OR ACCA OR PPP1R84 OR GPCR21 OR "G protein-coupled receptor 37 like 1" OR SREB1 OR 12-HETER OR HETER1 OR RVDR1 OR EDNRBL OR hET(B)R-LP OR PAELR OR ETBR-LP-2 OR GPR42P OR GPR41L OR FFAR3L OR PSP24 OR PSP24A OR H9 OR Mel1c OR BALGR OR PSP24B OR PSP24(beta) OR hTDAG8 OR TDAG8 OR OGR1 OR WI-31133 OR GPR79P OR GPR72 OR EX33 OR SREB2 OR GPR95 OR hGPCR2 OR GPCR2 OR G2A OR PAFR OR HUMNPIIY20 OR PGR3 OR PGR13 OR PGR2 OR PGR8 OR PGR6 OR PGR10 OR PGR11 OR PGR7 OR PGR5 OR PGR1 OR IEDA OR R35 OR GALR4 OR GPCR150 OR GPCR1 OR RE2 OR A-2 OR GRCA OR H963 OR SREB3 OR FKSG79 OR Gm1012 OR hrhAMR OR G10D OR AM-R OR ADMR OR "G protein-coupled receptor") *;* | | | | | | |  |  |
| #5 | (P2RX1 OR P2RX2 OR P2RX3 OR P2RX4 OR P2RX5 OR P2RX6 OR P2RX7 OR "purinergic receptor P2X 1" OR "purinergic receptor P2X 2" OR  "purinergic receptor P2X 3" OR "purinergic receptor P2X 4" OR "purinergic receptor P2X 5" OR "purinergic receptor P2X 6" OR "purinergic receptor P2X 7" OR P2X1 OR P2X2 OR P2X3 OR P2X4 OR P2X5 OR P2X6 OR P2X7 OR DFNA41 OR P2RXL1 OR LRH-1 OR P2XM OR MGC129625 OR MGC20089 OR "purinergic receptors" OR "ionotropic purinoceptors" OR GPR1 OR GPR3 OR GPR4 OR GPR6 OR GPR12 OR GPR15 OR GPR17 OR GPR18 OR GPR19 OR GPR20 OR GPR21 OR GPR22 OR GPR25 OR GPR26 OR GPR27 OR GPR31 OR GPR32 OR GPR33 OR GPR34 OR GPR35 OR GPR37 OR GPR37L1 OR GPR39 OR GPR42 OR GPR45 OR GPR50 OR GPR52 OR GPR55 OR GPR61 OR GPR62 OR GPR63 OR GPR65 OR GPR68 OR GPR75 OR GPR78 OR GPR79 OR GPR83 OR GPR82 OR GPR84 OR GPR85 OR GPR87 OR GPR88 OR GPR101 OR GPR119 OR GPR132 OR GPR135 OR GPR139 OR GPR141 OR GPR142 OR GPR146 OR GPR148 OR GPR149 OR GPR150 OR GPR151 OR GPR152 OR GPR153 OR GPR160 OR GPR161 OR GPR162 OR GPR171 OR GPR173 OR GPR174 OR GPR176 OR GPR183 OR GPR182 OR LGR4 OR LGR5 OR LGR6 OR MAS1 OR MAS1L OR MRGPRD OR MRGPRE OR MRGPRF OR MRGPRG OR MRGPRX1 OR MRGPRX2 OR MRGPRX3 OR MRGPRX4 OR P2RY8 OR P2RY10 OR "P2Y receptor family member 10" OR "P2Y receptor family member 8" OR P2Y10 OR P2Y8 OR MRGX4 OR MRGX3 OR MRGX2 OR MRGX1 OR "MAS related GPR family member X4” OR "MAS related GPR family member X3" OR "MAS related GPR family member X2" OR "MAS related GPR Family member X1" OR "MAS related GPR family member G" OR "MAS related GPR Family  member F" OR "MAS related GPR family member E" OR "MAS related GPR family member D" OR GPR169 OR GPR168 OR GPR167 OR GPR140 OR mrgG OR mrgF OR mrgE OR mrgD OR MGC21621 OR dJ994E9.2 OR MRG OR MAS-L OR "MAS1 proto-oncogene like, G protein-coupled receptor" OR "MAS1 proto-oncogene, G protein-coupled receptor" OR "leucine rich repeat containing G protein-coupled receptor 6" OR "leucine rich repeat containing G protein-coupled receptor 5" OR "leucine rich repeat containing G protein-coupled receptor 4") | | | | | | |  |  |
| #4 | (KCNK1 OR KCNK2 OR KCNK3 OR KCNK4 OR KCNK5 OR KCNK6 OR KCNK7 OR KCNK9 OR KCNK10 OR KCNK12 OR KCNK13 OR KCNK15 OR KCNK16 OR KCNK17 OR KCNK18 OR "potassium two pore domain channel subfamily K member 1" OR "potassium two pore domain channel  subfamily K member 2" OR "potassium two pore domain channel subfamily K member 3" OR "potassium two pore domain channel subfamily K member 4" OR "potassium two pore domain channel subfamily K member 5" OR "potassium two pore domain channel subfamily K member 6" OR “potassium two pore domain channel subfamily K member 7" OR "potassium two pore domain channel subfamily K member 9" OR "potassium two pore domain channel subfamily K member 10" OR "potassium two pore domain channel subfamily K member 12" OR "potassium two pore domain channel subfamily K member 13" OR "potassium two pore domain channel subfamily K member 15" OR "potassium two pore domain channel subfamily K member 16" OR "potassium two pore domain channel subfamily K member 17" OR "potassium two pore domain channel subfamily K member 18" OR KCNK11 OR KCNK14 OR K2p1.1 OR K2p2.1 OR DPK OR TWIK-1 OR TREK-1 OR K2p3.1 OR TASK OR TASK-1 OR K2p4.1 OR TRAAK OR K2p5.1 OR TASK-2 OR K2p6.1 OR TWIK-2 OR K2p9.1 OR K2p7.1 OR TASK3 OR TASK-3 OR K2p10.1 OR TREK-2 OR TREK2 OR PPP1R97 OR THIK-2 OR THIK2 OR K2p12.1 OR K2p13.1 OR THIK-1 OR THIK1 OR K2p15.1 OR dJ781B1.1 OR KT3.3 OR KIAA0237 OR TASK5 OR TASK-5 OR K2p16.1 OR TALK-1 OR TALK1 OR K2p17.1 OR TALK-2 OR TALK2 OR TASK4 OR TASK-4 OR K2p18.1 OR TRESK-2 OR TRESK2 OR TRESK OR TRIK OR "two-pore-domain potassium channel") | | | | | | |  |  |
| #3 | (TRPA1 OR "transient receptor potential cation channel subfamily A member 1" OR ANKTM1 OR TRPC1 OR "transient receptor potential  cation channel subfamily C member 1" OR HTRP-1 OR TRPC2 OR "transient receptor potential cation channel subfamily C member 2, pseudogene" OR TRPC3 OR "transient receptor potential cation channel subfamily C member 3" OR "transient receptor potential cation channel subfamily C member 4" OR "transient receptor potential cation channel subfamily C member 5" OR "transient receptor potential cation channel subfamily C member 6" OR "transient receptor potential cation channel subfamily C member 7" OR TRPC4 OR TRPC5 OR TRPC6 OR TRPC7 OR FSGS2 OR HTRP4 OR TRP4 OR PPP1R159 OR TRP6 OR MCOLN1 OR MCOLN2 OR MCOLN3 OR mucolipin 1 OR mucolipin 2 OR mucolipin 3 OR TRPM-L1 OR MSTP080 OR MST080 OR MLIV OR TRPML1 OR ML4 OR FLJ36691 OR TRP-ML2 OR TRPML2 OR TRP-ML3 OR TRPML3 OR FLJ11006 OR TRPM1 OR TRPM2 OR TRPM3 OR TRPM4 OR TRPM5 OR TRPM6 OR TRPM7 OR TRPM8 OR "transient receptorpotential cation channel subfamily M member 1" OR "transient receptor potential cation channel subfamily M member 2" OR "transient receptor potential cation channel subfamily M member 3" OR "transient receptor potential cation channel subfamily M member 4" OR "transient receptor potential cation channel subfamily M member 5) OR "transient receptor potential cation channel subfamily  M member 6” OR "transient receptor potential cation channel subfamily M member 7" OR "transient receptor potential cation channel subfamily M member 8" OR MLSN1 OR LTRPC1 OR CSNB1C OR TRPC7 OR KNP3 OR LTRPC2 OR NUDT9L1 OR NUDT9H OR EREG1 OR KIAA1616 OR LTRPC3 OR GON-2 OR FLJ20041 OR LTRPC5 OR MTR1 OR HOMG OR HSH OR CHAK2 OR FLJ22628 OR CHAK1 OR LTRPC7 OR TRP-PLIK OR PKD2 OR PKD2L1 OR PKD2L2 OR "polycystin 2, transient receptor potential cation channel" OR "polycystin 2 like 1, transient receptor potential cation channel" OR "polycystin 2 like 2, transient receptor potential cation channel" OR PKD2L OR PKDL OR TRPP3 OR PCL OR TRPP5 OR VR1 OR TRPV1 OR TRPV2 OR TRPV3 OR TRPV4 OR TRPV5 OR TRPV6 OR "transient receptor potential cation channel subfamily V member 1” OR "transient receptor potential cation channel subfamily V member 2" OR "transient receptor potential cation channel subfamily V member 3" OR "transient receptor potential cation channel subfamily V member 4" OR "transient receptor potential cation channel subfamily V member 5" OR "transient receptor potential cation channel subfamily V member 6" OR VRL OR VRL-1 OR VRL1 OR VRL3 OR OTRPC4 OR TRP12 OR VROAC OR VRL-2 OR VR-OAC OR CMT2C OR ECAC1 OR CaT2 OR ECAC2 OR CaT1 OR Transient receptor potential channels OR TRP channels OR "vanilloid receptor subtype 1" OR "amiloride-sensitive cation channel 1, neuronal" OR ""amiloride-sensitive cation channel" OR acid chemosensors) | | | | | | |  |  |
| #2 | ("acid sensing ion channels" OR ASIC* OR ASIC2 OR "acid-sensing ion channels" OR ASIC 2 OR ASIC3 OR ASIC 3 OR ASIC1 OR ASIC 1 OR ASIC4  OR ASIC 4 OR ASIC5 OR ASIC 5 OR BNaC2 OR hBNaC2 OR ASIC2a OR BNC1 OR BNaC1 OR hBNaC1 OR MDEG OR TNaC1 OR DRASIC OR BNAC4 OR INAC OR HINAC OR ACCN2 OR ACCN OR ACCN1 OR ACCN3 OR ACCN4 OR ACCN5 OR "acid sensing ion channel subunit 1" OR "acid sensing ion channel subunit 2" OR "acid sensing ion channel subunit 3" OR "acid sensing ion channel subunit family member 4" OR "acid sensing ion channel subunit family member 5”) | | | | | |  | |  |
| #1 | (microglia* OR gliosis OR glia* OR nissl OR macrophage* OR monocyte* OR CD11 OR CD68 OR CD40 OR CD45 OR Ox-42 OR Ox42 OR ed-1 OR ed1 OR cd200 OR cd 200 OR Iba1 OR Iba1 OR ly6g OR cd3 OR mpo OR mcp1 OR mcp-1 OR ccr2 OR arg1 OR arg 1 OR mhc OR major histocompatibility complex OR aldh1 OR aldh 1 OR hla dr OR cd20 OR HLA OR neuroglia OR leukocytes OR antibody-producing cells OR  antigen-presenting cells OR neurogenic inflammation OR astrocyte*) | | | | | |  | |  |

**Pubmed– December 31^th^ 2017 - 2245 references**


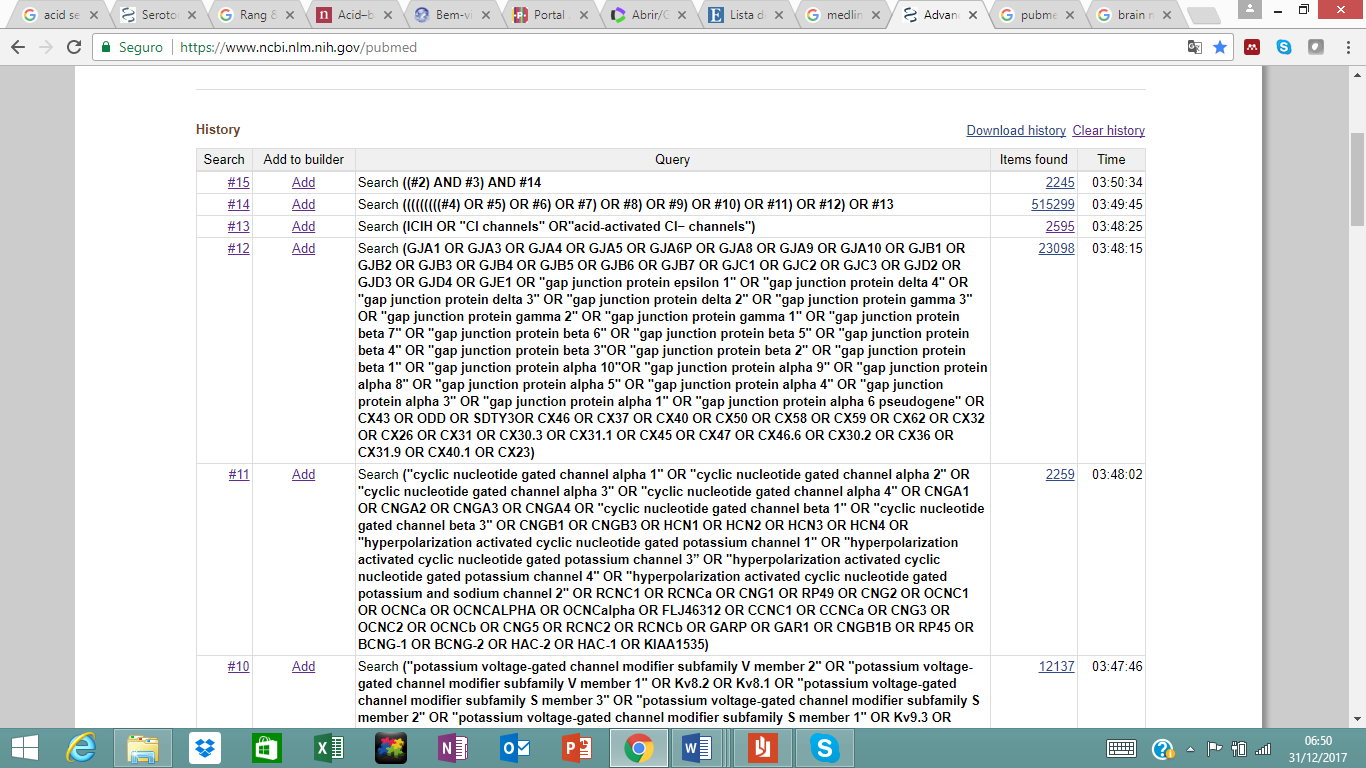


**Web of Science -December 31^th^ 2017– 4117 references**


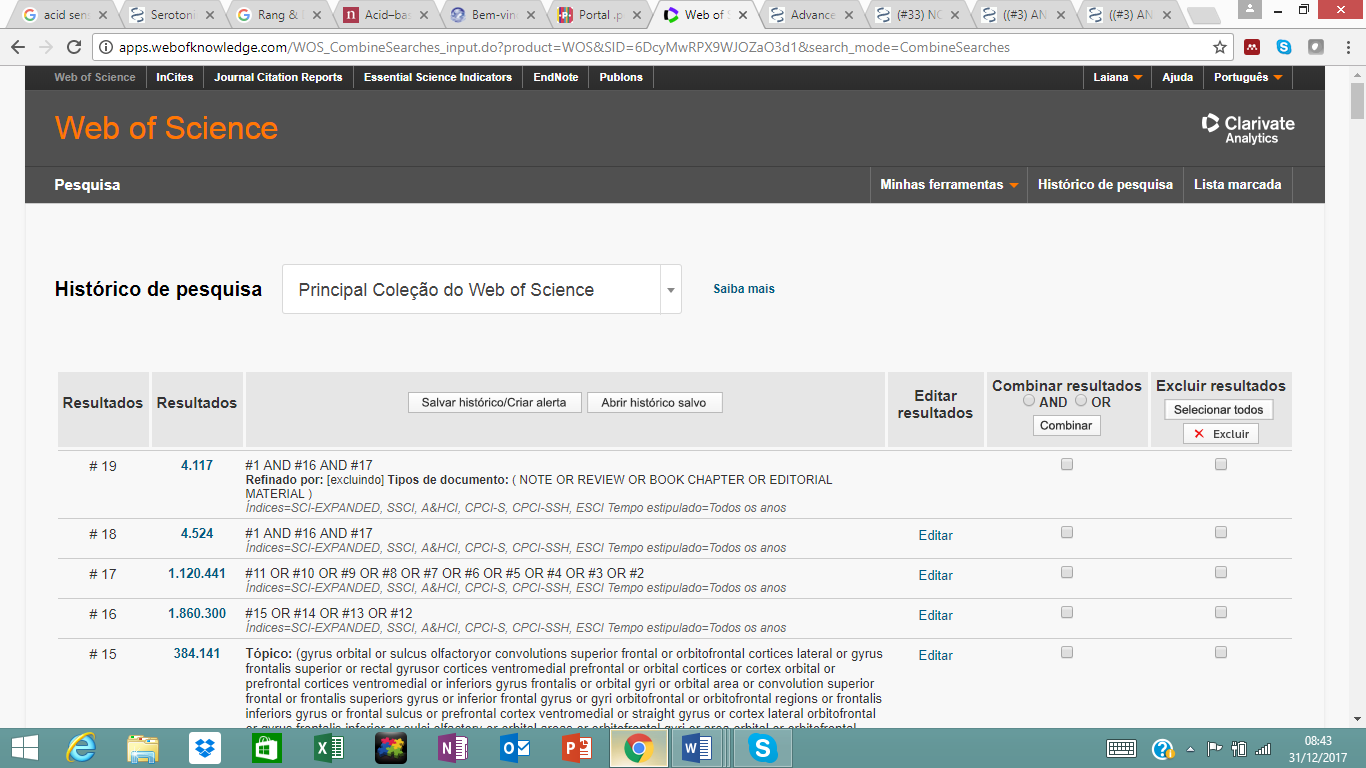


**Appendix 3: Custom data extraction form used for included studies**

| **Study ID:** | | |  |  |  |
| --- | --- | --- | --- | --- | --- |
| **Coder initials:** | | |  |  |  |
| **Date started:** | | |  |  |  |
| **Date completed:** | | |  |  |  |
| **Title of the paper:** | | |  |  |  |
| **Year of publication:** | | |  |  |  |
| **Journal:** | | |  |  |  |
| **Location (country):** | | |  |  |  |
| **Study design** | | |  |  |  |
| **1. Randomised controlled trial** | | |  |  |  |
| **2. Controlled trial / pseudo-randomised / quasi-experimental** | | |  |  |  |
| **3. Cohort** | | |  |  |  |
| **4. Clinical audit** | | |  |  |  |
| **5. Case-control** | | |  |  |  |
| **6. Case series** | | |  |  |  |
| **7. Case report** | | |  |  |  |
| **8. Other design (specify)** |  | |  |  |  |
| **Population description (Strain):** |  | |  |  |  |
| **Sample size:** |  | |  |  |  |
| **Sex of animals** |  | |  |  |  |
| ***n* (males)** |  | |  |  |  |
| ***n* (females)** |  | |  |  |  |
| **Age of animals** |  | |  |  |  |
| **Mean** |  | |  |  |  |
| **Range** |  | |  |  |  |
| **Primary aim of study:** |  | |  |  |  |
| **Intervention / level of exposure** | **Prescription** | | **Reported** |  |  |
| **Number of participants** |  |  |  |  |  |
| **Duration of treatment** |  |  |  |  |  |
| **How did the study define the intervention?** |  |  |  |  |  |
| **How did the study deliver the intervention?** |  |  |  |  |  |
| **Antagonist characteristics** |  |  |  |  |  |
| **Total daily dosage** |  |  |  |  |  |
| **Other treatment (additional to antagonists of ion channels)** |  |  |  |  |  |
| **Type of comparison** | | |  |  |  |
| **Microglia activate vs. microglia at a resting state** | | |  |  |  |
| **Comparator** | **Prescription** | | **Reported** |  |  |
| **Number of participants** |  |  |  |  |  |
| **Duration of treatment** |  |  |  |  |  |
| **How did the study define the comparator?** |  |  |  |  |  |
| **How did the study deliver the comparator?** |  |  |  |  |  |
| **Total daily antagonist dosage** |  |  |  |  |  |
| **Other treatment (additional to the antagonist)** |  |  |  |  |  |
| **Outcome : microglia in an activate or at resting state.** |  |  |  |  |  |
| **Is outcome tool validated?** |  |  |  |  |  |
| **Time points measured** |  |  |  |  |  |
| **Between Groups** |  |  |  |  |  |
| **Effect size (difference between groups)** |  |  |  |  |  |
| **Effect size (%) (if different units)** |  |  |  |  |  |
| **Level of significance (P-value or CI)** |  |  |  |  |  |
| **Direction of result** |  | **1. Favours intervention** | **2. Favours comparator** |  |  |
| **Sponsorship / funding (verbatim):** |  |  |  |  |  |
| **Authors conflicts of interest** |  |  |  |  |  |
|  | | |  |  |  |

**Appendix 5: Supplementary table 1. Quality score.**

|  | **1** | **2** | **3** | **4** | **5** | **6** | **7** | **8** | **9** | **10** |
| --- | --- | --- | --- | --- | --- | --- | --- | --- | --- | --- |
| **Study** | **Selection bias 1** | **Selection bias 2** | **Selection bias 3** | **Performance bias 1** | **Performance bias 2** | **Detection bias 1** | **Detection bias 2** | **Attrition bias** | **Reporting bias** | **Other potential bias** |
| Choi et al. 2012 | ? | ✓ | ✓ | ? | x | x | x | ✓ | ✓ | ✓ |
| Huang et al. 2017 | x | ✓ | ✓ | ? | x | ✓ | x | ✓ | ✓ | ✓ |
| Lee et al. 2014 | ✓ | ✓ | ✓ | ✓ | ✓ | ✓ | ✓ | ✓ | ✓ | ✓ |
| Yu et al. 2013 | x | ✓ | ✓ | ? | x | x | x | ✓ | ✓ | ✓ |
| Chu et al. 2012 | x | ✓ | ✓ | ? | x | x | x | ✓ | ✓ | ✓ |
| Melani et al. 2006 | ✓ | ✓ | ✓ | ✓ | x | ✓ | x | ✓ | ✓ | ✓ |
| Wixey et al. 2009 | ✓ | ✓ | ✓ | ✓ | x | ✓ | x | ✓ | ✓ | ✓ |
| Ortega et al. 2012 | ✓ | ✓ | ✓ | ✓ | ✓ | ✓ | ✓ | ✓ | ✓ | ✓ |
| Gelosa et al. 2014 | ✓ | ✓ | ✓ | ✓ | ✓ | ✓ | ✓ | ✓ | ✓ | ✓ |
| Liu et al. 2017 | ✓ | ✓ | ✓ | ✓ | x | ✓ | x | ✓ | ✓ | ✓ |
| Zhou et al. 2014 | ✓ | ✓ | ✓ | ✓ | ✓ | ✓ | ✓ | ✓ | ✓ | ✓ |
| He et al. 2012 | ✓ | ✓ | ✓ | ✓ | x | ✓ | x | ✓ | ✓ | ✓ |
| Wang et al. 2017 | ✓ | ✓ | ✓ | ✓ | ✓ | ✓ | ✓ | ✓ | ✓ | ✓ |
| Wu et al. 2016 | ✓ | ✓ | ✓ | ✓ | x | ✓ | x | ✓ | ✓ | ✓ |
| Choi et al. 2007 | x | ✓ | ✓ | ? | x | x | x | ✓ | ✓ | ✓ |

**Appendix 6: Supplementary table 2. Additional information about included studies**

| **Study** | **species** | **sex** | **weight** | **age** |
| --- | --- | --- | --- | --- |
| Choi et al. 2012 | Sprague-Dawle rats | male | 260-320 | 9-11 weeks |
| Huang et al. 2017 | Sprague- Dawle rats | male | 280-320 g | na |
| Lee et al. 2014 | Sprague- Dawle rats | male | na | 7 weeks |
| Yu et al. 2013 | Sprague- Dawle rats | male | 250 -300 g | na |
| Chu et al. 2012 | Sprague- Dawle rats | male | 260 - 320 g | na |
| Melani et al. 2006 | Wistar rats | male | 270 -290 g | na |
| Wixey et al. 2009 | Sprague- Dawle rats | mixed sexes | na | Post-natal day 3 |
| Ortega et al. 2012 | Wistar rats | male | 250 - 300 g | 3 months |
| Gelosa et al. 2014 | Sprague- Dawle rats | male | 200 - 250 g | na |
| Liu et al. 2017 | Sprague–Dawley rats | male | 250-300 g | na |
| Zhou et al. 2014 | case control | Sprague- Dawle rats | 180-220 g | na |
| He et al. 2012 | Sprague- Dawle rats | na | 180-220 g | na |
| Wang et al. 2015 | Sprague- Dawle rats | male | 250 - 300 g | na |
| Choi et al. 2007 | Sprague- Dawle rats | male | 240 - 260 g | na |
| Wu et al. 2016 | C57BL/6J and BALB/c mice | mixed sexes | na | 3, 6, 9, and 12 months |
